# Anti-tubercular activity and molecular docking studies of indolizine derivatives targeting mycobacterial InhA enzyme

**DOI:** 10.1080/14756366.2021.1919889

**Published:** 2021-07-01

**Authors:** Katharigatta N. Venugopala, Sandeep Chandrashekharappa, Pran Kishore Deb, Christophe Tratrat, Melendhran Pillay, Deepak Chopra, Nizar A. Al-Shar’i, Wafa Hourani, Lina A. Dahabiyeh, Pobitra Borah, Rahul D. Nagdeve, Susanta K. Nayak, Basavaraj Padmashali, Mohamed A. Morsy, Bandar E. Aldhubiab, Mahesh Attimarad, Anroop B. Nair, Nagaraja Sreeharsha, Michelyne Haroun, Sheena Shashikanth, Viresh Mohanlall, Raghuprasad Mailavaram

**Affiliations:** a Department of Pharmaceutical Sciences, College of Clinical Pharmacy, King Faisal University, Al-Ahsa, Saudi Arabia; b Department of Biotechnology and Food Technology, Durban University of Technology, Durban, South Africa; c Institute for Stem Cell Science and Regenerative Medicine (inStem), Bangalore, India; d Faculty of Pharmacy, Department of Pharmaceutical Sciences, Philadelphia University, Amman, Jordan; e Department of Microbiology, National Health Laboratory Services, KZN Academic Complex, Inkosi Albert Luthuli Central Hospital, Durban, South Africa; f Department of Chemistry, Indian Institute of Science Education and Research Bhopal, Bhopal, India; g Faculty of Pharmacy, Department of Medicinal Chemistry and Pharmacognosy, Jordan University of Science and Technology, Irbid, Jordan; h Department of Pharmaceutical Sciences, School of Pharmacy, The University of Jordan, Amman, Jordan; i Pratiksha Institute of Pharmaceutical Sciences, Guwahati, India; j Department of Chemistry, Visvesvaraya National Institute of Technology, Nagpur, India; k Department of Chemistry, School of Basic Science, Rani Channamma University, Belagavi, India; l Faculty of Medicine, Department of Pharmacology, Minia University, El-Minia, Egypt; m Department of Pharmaceutics, Vidya Siri College of Pharmacy, Bangalore, India; n Department of Studies in Organic Chemistry, University of Mysore, Mysore, India; o Pharmaceutical Chemistry Division, Sri Vishnu College of Pharmacy, Bhimavaram, India

**Keywords:** Indolizine, mycobacterium tuberculosis, InhA, docking, X-ray crystal structure

## Abstract

A series of 1,2,3-trisubstituted indolizines (**2a–2f, 3a–3d**, and **4a–4c**) were screened for *in vitro* whole-cell anti-tubercular activity against the susceptible H37Rv and multidrug-resistant (MDR) *Mycobacterium tuberculosis* (MTB) strains. Compounds **2b–2d**, **3a–3d**, and **4a–4c** were active against the H37Rv-MTB strain with minimum inhibitory concentration (MIC) ranging from 4 to 32 µg/mL, whereas the indolizines **4a–4c,** with ethyl ester group at the 4-position of the benzoyl ring also exhibited anti-MDR-MTB activity (MIC = 16–64 µg/mL). *In silico* docking study revealed the enoyl-acyl carrier protein reductase (InhA) and anthranilate phosphoribosyltransferase as potential molecular targets for the indolizines. The X-ray diffraction analysis of the compound **4b** was also carried out. Further, a safety study (*in silico* and *in vitro*) demonstrated no toxicity for these compounds. Thus, the indolizines warrant further development and may represent a novel promising class of InhA inhibitors and multi-targeting agents to combat drug-sensitive and drug-resistant MTB strains.

## 1. Introduction

Tuberculosis (TB) is a communicable infectious disease and a major cause of illness, particularly in low-income countries. It is caused by the opportunistic bacillus *Mycobacterium tuberculosis* (MTB) which primarily attacks the lungs (pulmonary) but may later affect other parts (extra-pulmonary) of the body^1^. According to the World Health Organisation (WHO), TB is considered as one of the top 10 causes of death worldwide, and the leading cause of death from a single infectious agent^1^. In 2019, TB resulted in nearly 1.4 million deaths, including 208,000 deaths among human immunodeficiency virus (HIV) positive patients^2^. HIV-infected patients are 19 times more likely to develop TB than HIV-negative subjects^3^^,^^4^. Several factors have contributed to the continuous health threat of TB globally. This includes the development of drug resistance such as multidrug-resistant tuberculosis (MDR-TB), extensively drug-resistant tuberculosis (XDR-TB)^5^, and totally drug-resistant tuberculosis (TDR-TB)^6^; the co-morbidities with acquired immunodeficiency syndrome (AIDS)^7^^,^^8^ and the risks involved in developing diabetes mellitus among TB patients^9^^,^^10^.

**Figure 1.**
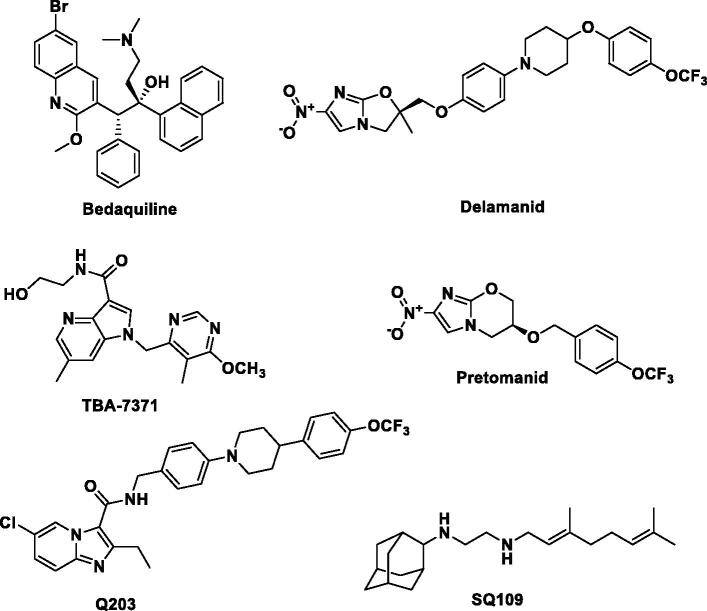
Chemical structures of approved anti-TB drugs (Bedaquiline, Delamanid and Pretomanid) and anti-TB compounds undergoing clinical trial (Q203, TBA-7371 and SQ109).

Several therapeutics have been approved by the US Food and Drug Administration (US-FDA) to enhance the treatment of TB. Bedaquiline (approved in late 2012 under the FDA’s accelerated review program) (Figure 1) was found to exhibit favourable anti-TB effects and promising anti-TB action against MDR and XDR-TB when combined with first-line or second-line anti-TB drugs, resulting in a shorter duration of treatment. However, bedaquiline suffered from various adverse effects such as nausea, elongation of QT interval, and drug interaction with Cytochrome P3A4 inducers and inhibitors^11^. Delamanid (Figure 1) was the second anti-TB agent approved by European Medicine Agency in late 2013. Nevertheless, resistant MTB strains against both bedaquiline and delamanid have recently been reported, which acquired resistance as a result of prolonged duration of therapy^12^^,^^13^. Pretomanid was the last drug candidate from the TB drug pipeline to be approved by the FDA in 2019 for the treatment of MDR and XDR TB (Figure 1)^14^^,^^15^.

For more than 40 years, there has been no significant active research in the arena of anti-TB drug discovery despite the fact that currently available drugs are considered cytotoxic and have low efficacy due to the emergence of resistant bacterial strains^16^. A recent call for the development of novel potential therapeutic agents to overcome MDR, XDR and TDR-TB have led to the discovery of a limited number of anti-TB drug candidates^17^. Among them, TBA-7371 (an azaindole) and Q203 or telacebac (an imidazopyridine amide) are very promising drug candidates for the potential treatment of MDR and XDR TB (Figure 1). However, considering the high attrition rate of drug candidates during clinical development, there is an urgent need to intensify and broaden the scope of the search for novel anti-TB lead structures by utilising validated new mycobacterial drug targets besides those targeted by current anti-TB therapies. A promising emerging approach to overcome MDR-TB is the development of multi-targeting compounds in which a single molecule has the ability to bind to different biological targets^18^. This concept is known as polypharmacology and has been proven promising in terms of efficacy, synergistic effect, and adverse events, and in preventing both drug-drug interaction and resistance insurgence^18^. For instance, SQ109, a drug candidate in the TB drug development pipeline, exhibits its anti-TB activity by acting on multiple targets (Figure 1). SQ109 is a well-known MmpL3 inhibitor that also shows inhibitory activity against MenA and MenG MTB enzymes as well^19^. It is worth mentioning that recently several natural products have also been reported as lead molecules against clinical MDR strains of MTB^20^. Therefore, the discovery of novel chemical entities having multiple modes of action is of paramount importance in the treatment of MDR, XDR, and TDR-TB infections.

**Figure 2.**
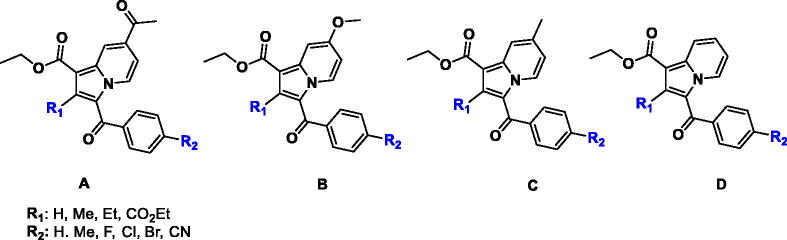
Indolizine structures with anti-TB activity developed by our group.

This study stems from our interest in designing new potential anti-TB therapeutics by focussing on structural modifications of the indolizine scaffold which has demonstrated potential anti-mycobacterial activity as previously reported by our group^21–23^ and other researchers^24–27^. Previously, we identified a series of poly-substituted indolizine A^21^, B^22^, and C^23^ as promising anti-TB agents against MDR-TB (Figure 2). Based on our previously reported structure-activity relationship (SAR), the nature and the position of substituents on the indolizine scaffold were found to be very sensitive to retain the anti-mycobacterial activity against susceptible MTB strain as well as rifampicin and isoniazid-resistant clinical isolates of MTB. Approximately, two-thirds of the synthesised indolizines A, B, and C displayed moderate to good potency against susceptible H37Rv MTB strain, whilst half of the active derivatives demonstrated good inhibitory activity against MDR-MTB strain. Indolizine represents a privileged scaffold for the development of bioactive compounds. Several synthetic indolizines have also been reported to possess a broad spectrum of pharmacological activities such as analgesic, anti-inflammatory, anticancer, antidiabetic, antihistaminic, COX-2 inhibition, antileishmanic, antimicrobial, antimutagenic, antioxidant, antiviral, larvicidal, herbicidal and α7 nAChR inhibitors, anti-Alzheimer, antischizophrenic, anticonvulsant and inhibitors of various enzymes^28–30^.

Despite the structural resemblance in the main bicyclic core of our designed compounds with Q203 and TBA-7371, to date, no indolizine-based anti-TB drug candidates have entered preclinical trials. With regards to the above considerations and as part of our continuous interest in the discovery of new potential anti-TB drugs^31–41^ we report herein the anti-mycobacterial activity of a series of 1,2,3-trisubstituted indolizines of type **D** with the aim of expanding the current knowledge on our previously reported SAR by exploring the influence of substituents at 1, 2 and 3-position of the indolizine scaffold that might play a critical role in the anti-TB activity against H37Rv strain ATCC 25177 and MDR strains of MTB. Furthermore, the potential mechanism of action of the title compounds is suggested based on their anti-tubercular profile in parallel with the molecular modelling analyses. In addition, the ADMET and toxicity characteristics of the most promising indolizines are also presented.

## 2. Materials and methods

### 2.1. Chemistry

The chemicals reported here were obtained from Sigma-Aldrich Co. (St. Louis, MO, USA), while the solvents were obtained from Millipore Sigma (Burlington, MA, USA). Thin-layer chromatography (TLC) using silica gel (Sigma-Aldrich Co.) on aluminium foil was employed to observe the chemical reactions; *n*-hexane and ethyl acetate (4:6) were used as solvents. The reactions were visualised under an ultraviolet (UV)-light/iodine chamber. B-545 was used to measure the melting points (Büchi, Labortechnik, Flawil, Switzerland). Fourier-transform infrared (FT-IR) spectra were recorded on a Shimadzu FT-IR spectrophotometer. Furthermore, ^1^H- and ^13^C-NMR spectra were recorded on Bruker AVANCE III 400 MHz instruments using DMSO-d6 as a solvent. Chemical shifts (*δ*) were recorded in parts per million (ppm) downfield from tetramethylsilane, while the coupling constants (*J*) were recorded in Hz. The splitting pattern was documented as follows: s, singlet; d, doublet; q, quartette; m, multiplet. Liquid chromatography–mass spectrometry (LC–MS; Agilent 1100 series) was used to measure the mass spectra in conjunction with the MSD, as well as 0.1% aqueous trifluoroacetic acid in an acetonitrile system on the C18-BDS column. Then, elemental analysis was carried out using the analyser FLASH EA 1112 CHN (Thermo Finnigan LLC, New York, NY, USA). A single-crystal X-ray diffraction study was performed using a Bruker KAPPA APEX II DUO diffractometer equipped with a CCD detector; monochromated Mo K*α* radiation (*λ* = 0.71073 Å) was used. Data collection was carried out at 173(2) K using an Oxford Cryostream cooling system featuring the Bruker Apex II software.

#### 2.1.1. General synthetic procedure for the preparation of 1-(2-(substitutedphenyl)-2-oxoethyl)pyridin-1-ium bromides (1a-f) and ethyl 3-(substitutedbenzoyl)-2-methylindolizine-1-carboxylates (2a–2f and 3a–3d)

Compounds **1a–1f**, **2a–2f**, and **3a–3d** were synthesised, purified by column chromatography, and well characterised by FT-IR, NMR, LC-MS, and elemental analysis as reported earlier from our research group^42^. The purity of the compounds was ≥99% as measured by HPLC.

#### 2.1.2. General procedure for the preparation of diethyl-3-(4-substitutedbenzoyl)indolizine-1,2-dicarboxylates (4a–4c)

To a stirred solution of 1-(2-(4-fluoro/chloro/nitro-phenyl)-2-oxoethyl)pyridin-1-ium bromide (**1a/1b/1d**) (0.0016 mol), in water (10 mL), was added substituted diethyl 2-butynedioate (0.0016 mol), stirred at 80 °C for 3 h. Completion of reaction was monitored on TLC. The reaction mixture was diluted with ethyl acetate. The organic layer was separated, washed with brine and dried under sodium sulphate. The crude compound was purified by recrystallization method using hexane and ethyl acetate to afford 69–83% yield of diethyl-3-(4-substitutedbenzoyl)indolizine-1,2-dicarboxylate (**4a–4c**). The characterisation details of title compounds (**4a–4c**) are reported below, and their spectral data are shown in Figures S1–S9.

##### 2.1.2.1. Diethyl-3-(4-fluorobenzoyl)indolizine-1,2-dicarboxylate (4a)

Appearance; Brown solid. Yield 72%, MP 138–139, FT-IR (KBr neat cm^−1^): 2985, 1733, 1695, 1635, 1608, 1228. ^1^H NMR (400 MHz, CDCl_3_) *δ* = 9.51–9.48 (d, *J* = 7.2 Hz, 1H), 7.78–7.70 (m, 3H), 7.16–7.11 (m, 2H), 6.82–6.77 (m, 1H), 4.36–4.30 (q, *J* = 7.2 Hz, 2H), 3.76–3.68 (q, *J* = 7.2 Hz, 2H), 1.37–1.33 (t, *J* = 7.2 Hz, 3H), 1.13–1.11 (t, *J* = 7.2 Hz, 3H). ^13^C-NMR (100 MHz CDCl_3_) *δ* = 183.77, 165.17, 164.12, 163.62, 163.23, 159.92, 141.51, 136.09, 136.02, 132.21, 131.59, 131.23, 129.89, 119.59, 115.62, 114.87, 110.18, 102.38, 97.63, 61.66, 60.28, 55.87, 14.32, 13.52. LC-MS (ESI, Positive): *m/z*: (M + H)^+^: 384.38: Anal. calculated for: C_21_H_18_FNO_5_; C, 65.79; H, 4.73; N, 3.65; Found; C, 65.41; H, 4.50; N, 3.55.

##### 2.1.2.2. Diethyl-3-(4-chlorobenzoyl)indolizine-1,2-dicarboxylate (4b)

Appearance; Brown solid. Yield 74%, MP 137–138, FT-IR (KBr neat cm^−1^): 2989, 1736, 1696, 1637, 1605, 1227. ^1^H NMR (400 MHz, CDCl_3_) *δ* = 9.53–9.49 (d, *J* = 7.2 Hz, 1H), 7.76–7.71 (m, 3H), 7.15–7.10 (m, 2H), 6.82–6.77 (m, 1H), 4.37–4.30 (q, *J* = 7.2 Hz, 2H), 3.72–3.68 (q, *J* = 7.2 Hz, 2H), 1.37–1.33 (t, *J* = 7.2 Hz, 3H), 1.13–1.10 (t, *J* = 7.2 Hz, 3H). ^13^C-NMR (100 MHz CDCl_3_) *δ* = 183.51, 165.34, 164.15, 163.59, 163.30, 160.21, 141.49, 136.07, 135.89, 132.23, 131.28, 131.16, 129.91, 119.48, 115.49, 114.99, 110.24, 102.74, 97.69, 61.78, 60.36, 55.71, 14.42, 13.83. LC-MS (ESI, Positive): *m/z*: (M + H)^+^: 400.2: Anal. calculated for: C_21_H_18_ClNO_2_; C, 63.09; H, 4.54; N, 3.50; Found; C, 62.91; H, 4.48; N, 3.45.

##### 2.1.2.3. Diethyl-3-(4-nitrobenzoyl)indolizine-1,2-dicarboxylate (4c)

Appearance; Light green colour. Yield 70%, MP 111–112, FT-IR (KBr neat cm^−1^): 2983, 1739, 1700, 1649, 1603, 1225. ^1^H-NMR (400 MHz CDCl_3_) *δ* = 9.49–9.47 (d, *J* = 7.2 Hz, 1H), 7.77–7.72 (m, 3H), 7.15–7.11 (m, 2H), 6.81–6.78 (m, 1H), 4.38–4.32 (q, *J* = 7.2 Hz, 2H), 3.75–3.69 (q, *J* = 7.2 Hz, 2H), 1.36–1.33 (t, J = 7.2 Hz, 3H), 1.13–1.11 (t, *J* = 7.2 Hz, 3H). ^13^C-NMR (100 MHz CDCl_3_) *δ* = 184.77, 166.12, 165.08, 163.59, 163.29, 159.91, 142.47, 137.04, 136.03, 132.21, 131.25, 131.18, 129.95, 119.52, 116.59, 114.93, 109.15, 103.44, 98.59, 61.58, 60.29, 55.87, 14.31, 13.72. LC-MS (ESI, Positive): *m/z*: (M + H)^+^: 411.2: Anal. calculated for: C_21_H_18_N_2_O_7_; C, 61.46; H, 4.42; N, 6.83; Found; C, 61.31; H, 4.30; N, 6.55.

### 2.2. Crystallography

Single-crystal X-ray diffraction data of the compound **4b** was collected on a Bruker KAPPA APEX II DUO diffractometer using graphite-monochromated Mo-Kα radiation (*χ* = 0.71073 Å). Data collection^43^ was carried out at 173(2) K. Temperature was controlled by an Oxford Cryostream cooling system (Oxford Cryostat). Cell refinement and data reduction were performed using the SAINT program^44^. The data were scaled and absorption correction was performed using SADABS^44^.

The structure was solved by direct methods using SHELXS-97^45^ and refined by the full-matrix least-squares method based on F^2^ using SHELXL-2014^46^. All non-hydrogen atoms were refined anisotropically. All hydrogen atoms, were placed in idealised positions and refined in riding models with *U_iso_* assigned 1.2 or 1.5 times the *U_eq_* of their parent atoms, and the C–H bond distances were constrained to 0.95 Å for aromatic hydrogens and 1.00 Å for methylene and methyl hydrogens. All geometrical calculations were done using PLATON^47^. The program WinGx^48^ was used to prepare molecular graphic images.

### 2.3. Hirshfeld surface analysis and energy framework calculation

The Hirshfeld surfaces analysis is a technique for visualisation and investigation to illustrate the nature of intermolecular interactions. The Hirshfeld surfaces and two-dimensional fingerprint plot for compound **4b** have been generated using the software *Crystal Explorer 17.5*. This software has been also used to assess the interaction energies of the compound **4b**.

### 2.4. Anti-tubercular activity

The antitubercular activity of the designed compounds **2a–2f**, **3a–3d**, and **4a–4c** were evaluated against two types of MTB strains, namely H37Rv and well characterised MDR strains using the colorimetric Resazurin Microplate Assay (REMA) method^49^. A 100 µL of Middelbrook 7H9 broth was aseptically prepared and dispensed in each of the wells of a 96 well flat-bottomed microtiter plate with lids (Lasec, South Africa). Each of the test compounds was accurately weighed, dissolved in the appropriate solvent, and filter sterilised using a 0.2 micron polycarbonate filter. Stock solutions of the test samples were aliquoted into cryovials and stored at −20 °C. A 100 µL of the test samples were added to each of the wells containing Middlebrook 7H9 broth supplemented with 0.1% Casitone, 0.5% glycerol, and 10% OADC (oleic acid, albumin, dextrose, and catalase). The test samples were then serially diluted two folds directly in the broth of the microtiter plate to the desired concentration ranging from 40–0.625 µg/mL.

Inoculums from clinical isolates were prepared fresh from Middlebrook 7H11 agar plates by scraping and re-suspending loopful of colonies into Middlebrook 7H9 broth containing glass beads. The inoculum turbidity was adjusted to a McFarland number 1 standard and further diluted to 1:10 in M7H9 broth prior to the addition of 100 µL to each of the test samples and drug-free wells. Growth control and a sterile control were also included for each isolate. Sterile M7H9 broth was added to all perimeter walls to avoid evaporation during the incubation. The plate was covered, sealed in a plastic bag, and incubated at 37 °C. After 8 days of incubation, 30 µL of 0.02% working solution of resazurin salt was inoculated into each microtiter well. The plates were then incubated overnight and read the following day. A positive reaction resulted in a colour change from blue to pink owing to the reduction of resazurin to rezarufin which confirmed MTB cell viability/growth and, hence, drug resistance. The MICs were defined as the minimum drug concentration to inhibit the growth of the organism with no colour changes present in the well.

### 2.5. Safety studies (in vitro)

The safety of the tested indolizines was evaluated by MTT assay. The MTT (3-(4,5-dimethylthiazol-2-yl)-2,5-diphenyltetrazolium bromide) cytotoxicity assay was used to evaluate the cytotoxic effect of the most promising compounds against peripheral blood mononuclear cells (PBMCs) according to the described protocol^50^. Cells were pipetted (90 µL of cell culture, 1 × 10^5^ cells/mL) into each well of 96-well microtiter plates, and the outer wells were filled with PBS (phosphate buffer saline) in order to prevent the medium from evaporation during incubation. Thereafter, plates were incubated at 37 °C for 24 h. Each well of the plate was then treated with 10 µL of the compounds (1000–5 µg/mL). In the control wells, the negative control DMSO (dimethyl sulfoxide) and media were added. Thereafter, the plates were incubated for 2 days at 37 °C in a humidified incubator that contained a 5% CO_2_ atmosphere. After the incubation time, 20 µL of MTT reagent (5 mg/mL) was further added to the individual well. The plate was then incubated for a further 4 h at 37 °C (5% CO_2_ incubator). The media was then removed, and an aliquot of 100 µL DMSO was added to each well in order to dissolve the formazan crystals that were formed in metabolically active cells. After that, plates were incubated for an extra hour. The absorbance of the formazan was evaluated at 590 nm using an ELISA plate reader (Thermo Scientific Multiskan GO).

### 2.6. Molecular modelling

Molecular docking of the designed compounds into the active site of proposed potential targets was performed using Accelrys Discovery Studio 4.0 (from BIOVIA Software Inc.). The X-ray crystal structures of mycobacterial enyol-ACP-reductase (InhA) and anthranilate phosphoribosyltransferase (trpD) enzymes in complex with their inhibitors (PDB accession codes 5G0S and 3R6C, respectively) were retrieved from the RSCB Protein Data Bank.

Crystal complexes were prepared using the clean protein tool and prepare protein protocol in DS to standardise atoms names, correct connectivity and bond order, protonate the ionisable residues at a pH of 7.4, add missing residues and correct incomplete ones, and remove water molecules. To validate the docking protocol, the co-crystallised ligand was extracted and re-docked into the active site of the enzyme to ensure proper definition of the binding site and to assess the accuracy of the docking algorithm in reproducing the pose of the co-crystallised ligand. C-Docker protocol was used to perform the docking studies of the tested indolizines into their potential targets. The ligands are docked into a rigid receptor while a set of ligand conformation is generated. Additional scoring functions PLP1, PLP2, Jain, and PMF were utilised to ensure the optimal ligand orientation into the active binding site of the target. The highest negative score of PLP1, PLP2, Jain, and PMF^51^^,^^52^, indicating the strongest receptor-ligand binding affinities, is considered for refining binding poses. The binding energy calculation was performed using C-Docker protocol and *in situ* ligand minimisation protocol.

### 2.7. ADMET calculation (in silico)

The ADMET Descriptors protocol and the Toxicity Prediction (TOPKAT) protocol in DS were used to calculate a range of absorption, distribution, metabolism, excretion, and toxicity (ADMET) related properties including, aqueous solubility (AS), blood–brain barrier (BBB) penetration, CYP2D6 inhibition, hepatotoxicity, human intestinal absorption (HIA), plasma protein binding (PPB), AlogP, and polar surface area (PSA); in addition, different toxicity parameters including rodent carcinogenicity (for both male and female rats and mice, CMR, CFR, CLM and CFM respectively), Ames mutagenicity (AM), skin irritation (SI), ocular irritancy (OI), aerobic biodegradability and developmental toxicity potential (AB) were also calculated.

## 3. Results and discussion

### 3.1. Chemistry

**Scheme 1.**
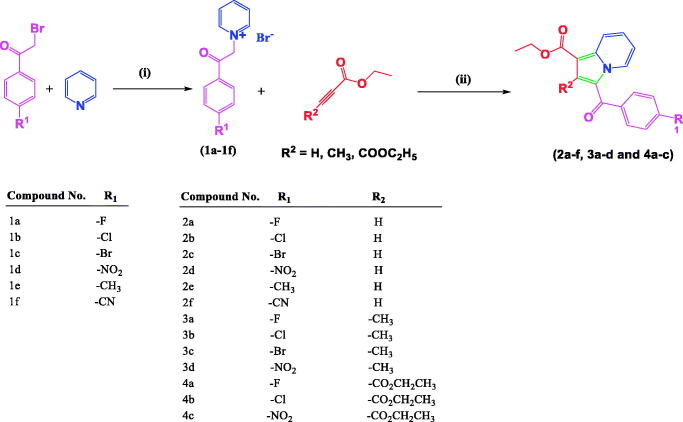
Synthesis of 1,2,3-trisubstiuted indolizine derivatives (**2a–2f**, **3a–3d**, **4a–4c**): Reagents and conditions (i) pyridine, dry acetone, stir at room temperature, 5 h; (ii) ethyl propialate/ethy1 2-butynoate/diethy1 2- butynedioate water, stir 80 °C, 3 h

The synthesis of the title compounds (**2a–2f**, **3a–3d**, **4a–4c**) is portrayed in Scheme 1. The intermediates (**1a–1f**) were obtained by stirring a mixture of pyridine, and para-substituted phenacyl bromides in a dry acetone medium at 5 h. Compounds **1a–1f** on further reaction with ethyl prop-2-ynoate/ethyl but-2-ynoate/diethyl 2-butynedioate in the presence of water with continuous stirring at 80 °C for 3 h resulted in the formation of title compounds **2a–2f**, **3a–3d**, **4a–4c**. The resulting title compounds were purified using ethyl acetate and hexane as an eluent by column chromatography, and the compound purity was found to be more than 99% with a satisfactory yield (69% to 83%). Synthesis, purification, and characterisation details of compounds **1a–1f**, **2a–2f**, and **3a–3d** were reported earlier from our research group^42^. The chemical structures of the newly synthesised compounds (**4a-4c**) have been ascertained with the help of spectroscopic techniques such as FT-IR, NMR (^1^H and ^13^C), LC-MS, and elemental analysis. In LC-MS, the molecular ion peaks of these novel compounds (**4a–4c**) were in good agreement with their proposed molecular masses. Elemental analysis results of the title compounds (**4a–4c**) were within ±0.4% of the calculated values.

The primary goal of the current study was to investigate the impact of the substitution pattern, in terms of nature and position of substituents, at positions 1, 2, and 3 of the indolizine ring system on the anti-tubercular activity of the resultant analogues. The synthetic strategy for the development of the target compounds involved a 1.3-dipolar [3 + 2] cycloaddition as a key step, allowing the introduction of the substituent in the diverse position of the indolizine ring. The 1.3-dipolar [3 + 2] cycloaddition of pyridinium ylides with electron-deficient alkynes offered a convenient approach for the construction of an indolizine scaffold.

### 3.2. Crystallography

**Figure 3.**
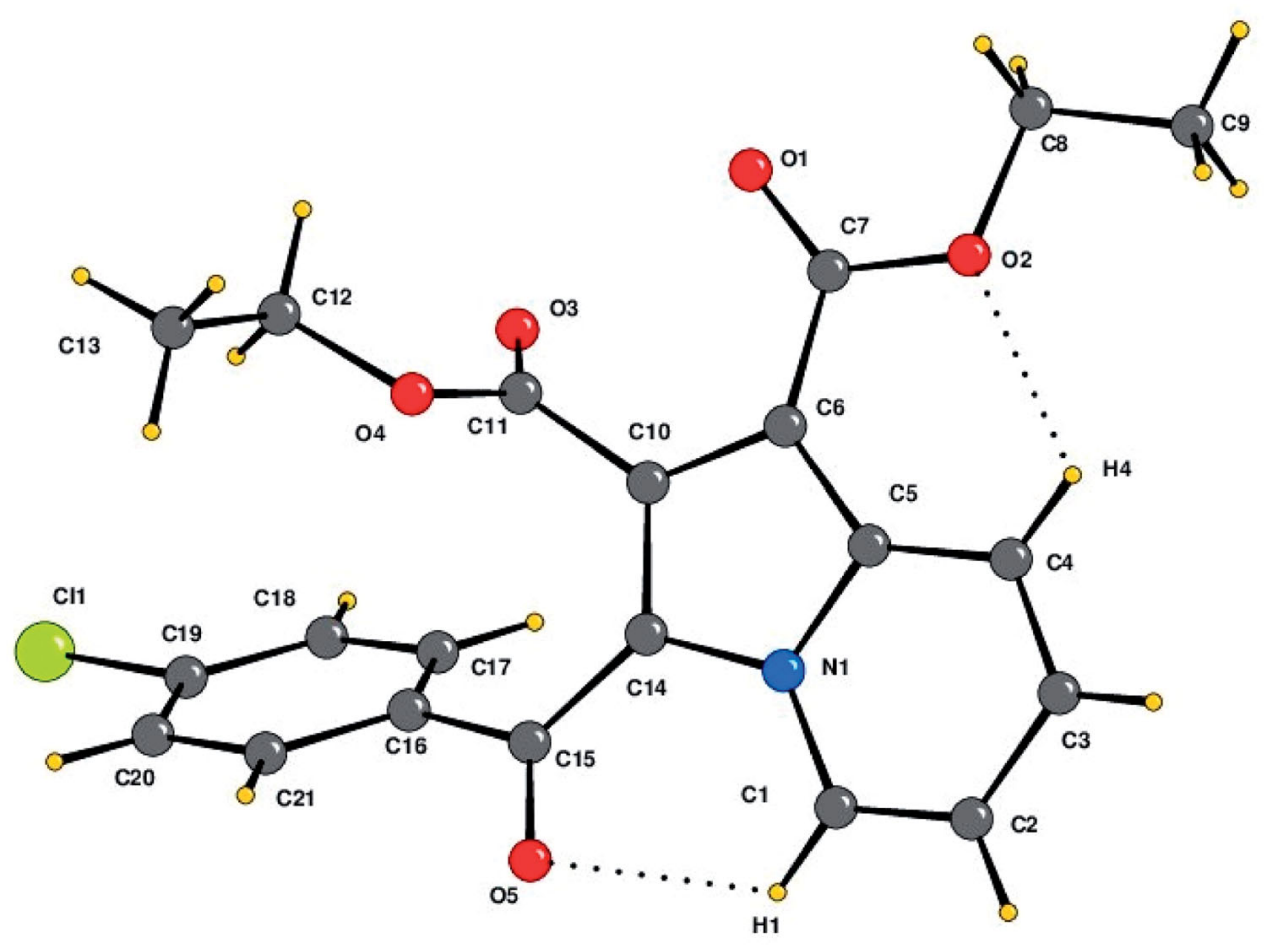
The crystal structure of diethyl 3-(4-chlorobenzoyl)indolizine-1,2-dicarboxylate (**4b**). ORTEP drawn with 50% ellipsoidal probability. Intra-molecular C–H···O·H bonds are shown as dotted lines.

**Figure 4.**
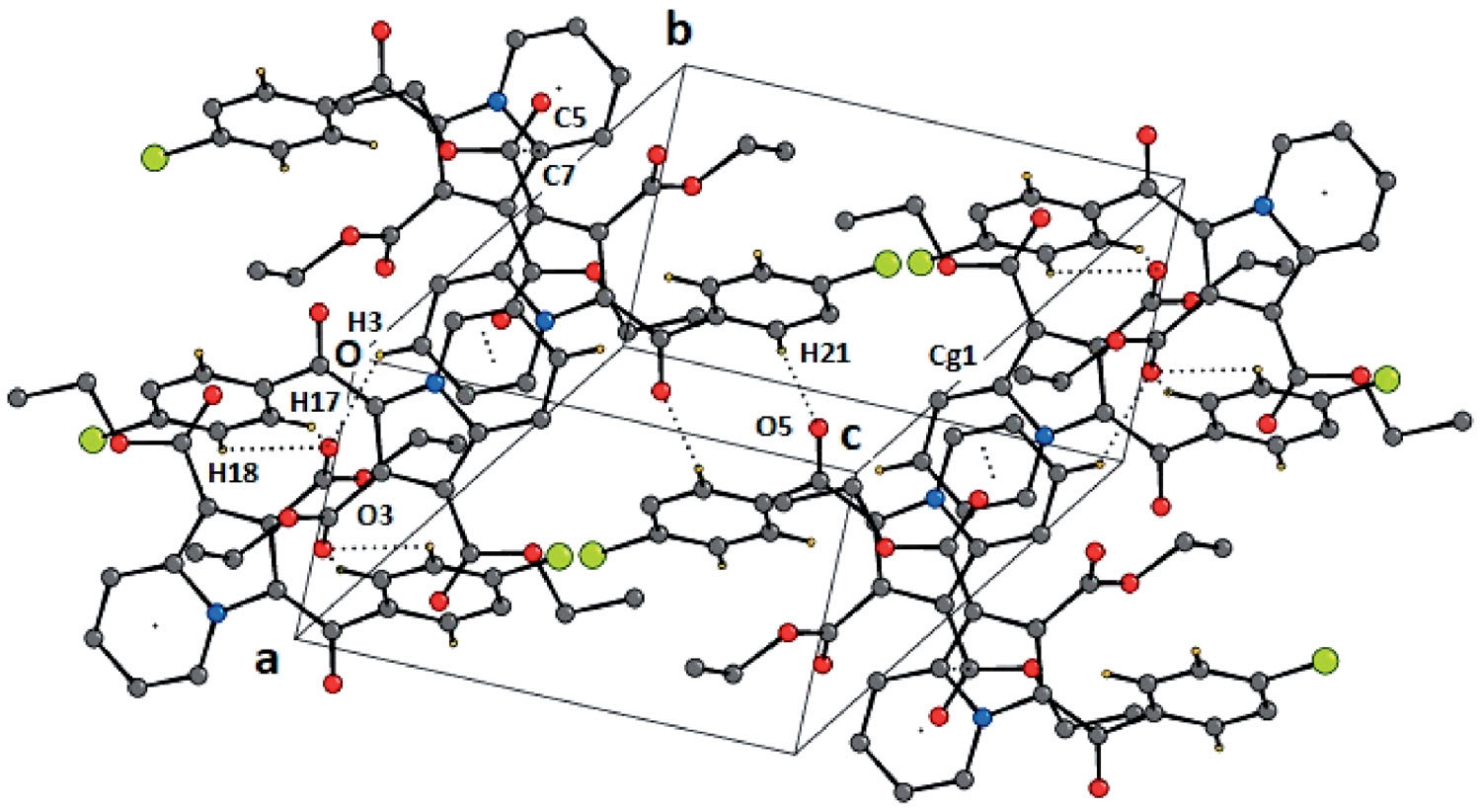
Crystal packing depicting C–H···O·H-bonds, π–π stacking interaction and O1 = C7···C5–N1 short contact in the compound **4b**. Cg1 is the centre of gravity of N1/C1–C5 ring. The dotted lines represent intermolecular interactions. Non-interacting hydrogens have been omitted for clarity.

Compound **4b** was crystallised in the triclinic centrosymmetric space group *P-1* with one molecule in the asymmetric unit. The parameters for crystal data collection and structure refinement, and the list of intramolecular and intermolecular interactions in the crystal structure of diethyl 3-(4-chlorobenzoyl)indolizine-1,2-dicarboxylate (**4b**) are given in Table S1 and Table 1, respectively. The side chain ethoxy groups do not exhibit any orientational disorder. The molecular conformation is locked via C-H···O H-bonds (involving H1/O5 and H4/O2) forming pseudo-six membered rings (Figure 3). The crystal packing is stabilised via C–H···O H-bonds (involving H21 and O5), forming dimers, and these are connected with centro symmetrically related dimers formed via C-H···O H-bonds (involving H17/H18 with O3) via additional C–H···O H-bonds (involving H3 and O3), thus leading to the overall formation of a “*hexamer*” in the solid-state (Figure 4). Thus, O3 functions as a “trifurcated acceptor” which is a unique feature in the packing of this molecule in the crystal. In addition, the molecules are connected via a π···π stacking motif, involving the 6-membered ring (Cg1: centre of gravity of ring N1/C1-C5), the stacking distance being 3.654 Å. Finally, a short contact between O1 = C7···C5–N1 of distance 3.359 Å, involving electrophilic C7 and relatively less electrophilic C5 is present in the crystalline lattice (Figure 4).

**Table 1.** List of intramolecular and intermolecular interactions in the crystal structure of diethyl 3–(4-chlorobenzoyl)indolizine-1,2-dicarboxylate (**4b**).

Interaction Type	D–H (Å)	D···A (Å)	H···A (Å)	D–H···A (°)	Symmetry code
C1–H1···O5	0.95	2.859	2.29	118	x, y, z
C4–H4···O2	0.95	2.869	2.33	115	x, y, z
C3–H3···O3	0.95	3.368	2.64	133	x, +y − 1, +z
C18–H18···O3	0.95	3.175	2.58	121	−x + 1, −y + 2, −z
C17–H17···O3	0.95	3.188	2.60	120	−x + 1, −y + 2, −z
C21–H21···O5	0.95	3.369	2.47	159	−x + 1, −y + 1, −z + 1

### 3.3. Hirshfeld surface analysis and energy framework calculation

**Figure 5.**
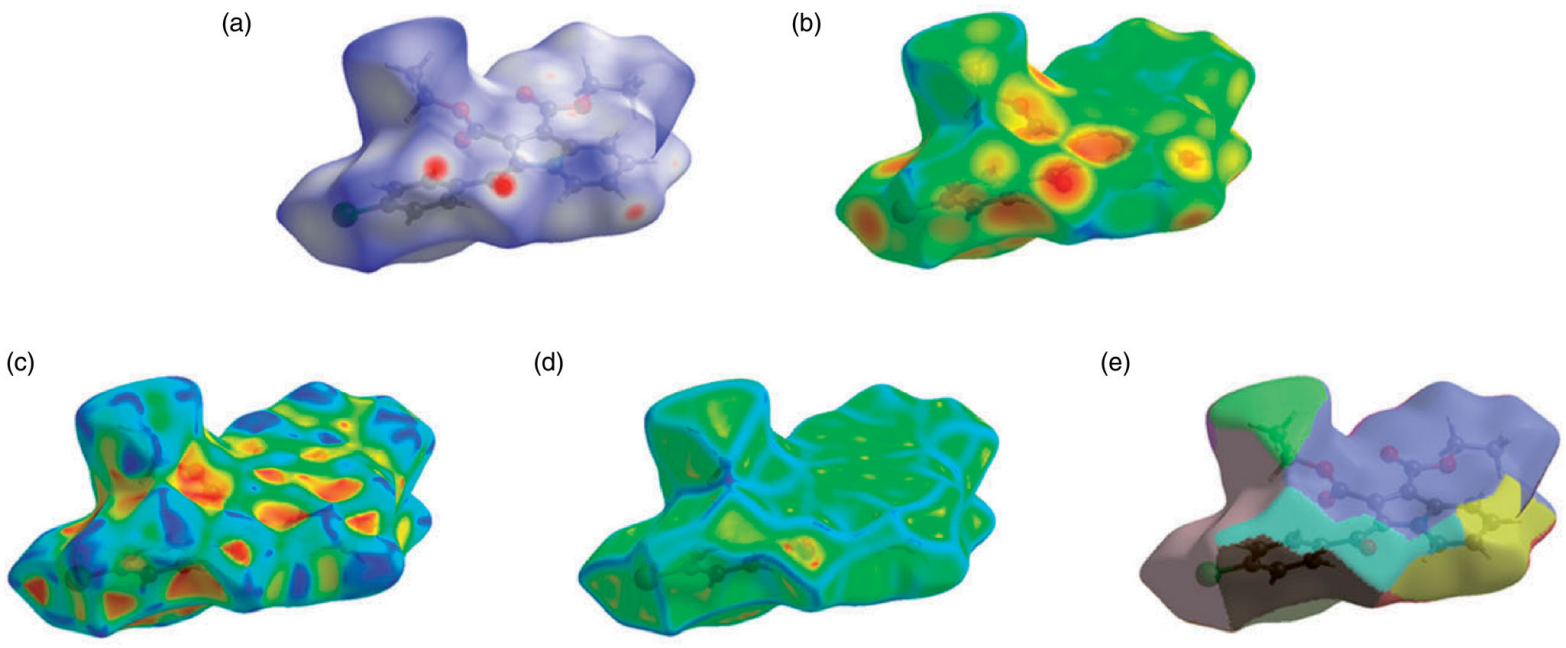
Hirshfeld surfaces mapped with (a) *d_norm_* (b) *d_e_* (c) shape-index, (d) curvedness and (e) fragment patches for the compound **4b**.

**Figure 6.**
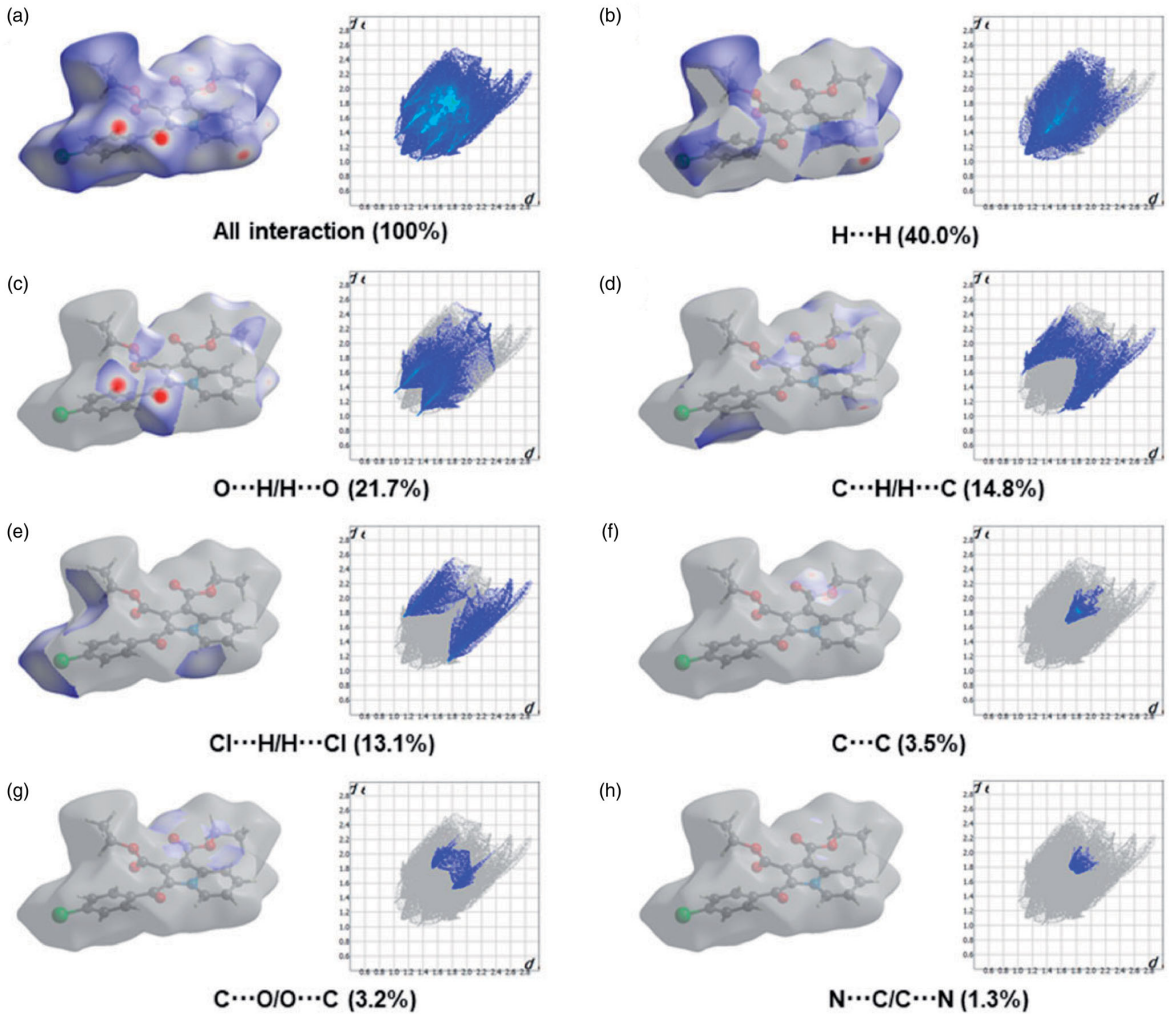
Two-dimensional fingerprint plot for the compound **4b** showing the contributions of individual types of interactions: (a) all intermolecular contacts, (b) H···H contacts, (c) O···H/H···O contacts, (d) C···H/H···C contacts, (e) Cl···H/H···Cl contacts, (f) C···C contacts, (g) C···O/O···C contacts, (h) N···C/C···N. The outline of the full fingerprint is indicating in grey. The related surface patches associated with the specific contacts with the *d_norm_* mapped are illustrated by surfaces to the left.

The Hirshfeld surfaces of the title compound **4b** were mapped over *d_e_*, *d_norm_*, shape index, curvedness, and fragment patches as shown in Figure 5. The contribution of individual intermolecular interactions on the Hirshfeld surface can be identified through the colour codes. If d_i_ is greater than d_e_, it is shown by the dark surface area that exists because of the acceptor of the hydrogen bond. The red colour spot indicates shorter molecular contacts on the *d_norm_* surface, and the blue colour on the surface of the *d_norm_* reflects longer molecular contacts. The white colour on the *d_norm_* surface indicates the interaction around the van der Waals radii. The red colour spot indicates the hydrogen bonding H···O contacts in the *d_norm_* surfaces, while the blue surface region represents the H···H contacts (Figure 5(a)). The characteristics of the d_e_ surface appear as a relatively flat green region where the distances of contact are similar (Figure 5(b)). The strong hydrogen bonding interactions present in the molecule (Figure 5(c)) are also demonstrated by the adjacent highlighted red and yellow regions on the shape index surface. Whereas, the H···H interactions (Figure 5(d)) are shown by the blue curved and yellow regions on the curvedness surfaces. The colour patches are mapped differently on the Hirshfeld surface depending on the closeness to the neighbouring molecules (Figure 5(e)). The 2D fingerprint plots indicate the sharp spike peak that reflects the strong hydrogen bonding present in the molecule (Figure 6(a–h)). The fingerprint plot shows that the H···H contacts have a comparatively larger contribution of 40.0% relative to other interactions and implies that van der Waals radii and hydrogen bonding play a key role in the crystal packing. The H···O contacts contribute 21.7% of the total interactions. In addition, the percentage contribution in this crystal structure of other intermolecular interactions are as follows: C···H/H···C (14.8%), H···Cl/Cl···H (13.1%), C···C (3.5%), C···O/O···C (3.2%), N···C/C···N (1.3%), O···Cl/Cl···O (1.1%), N···H/H···N (0.7%), O···N/N···O (0.4%), Cl···C/C···Cl (0.1%).

Systematic and theoretical energy was measured in terms of electrostatic, dispersion, and total energy using the program Crystal Explorer software, which further facilitated to depict the 3D topological images for the compound **4b** (detailed information is provided in the supplementary section Figures S10-S13 and Table S2).

### 3.4. Anti-tubercular activity

The anti-tubercular activity of the target compounds (**2a–2f**, **3a–3d**, and **4a–4c**) was evaluated (*in vitro*) against two different MTB strains, namely susceptible H37Rv MTB strain, and rifampicin and isoniazid-resistant MTB strain (Table 2). Rifampicin and isoniazid were also used as positive controls (Table 2). The anti-mycobacterial activity showed that compound **3a**, substituted with a fluorine atom at the 4-position of the benzoyl group and a methyl group at the 2-position of the indolizine ring, is the most potent molecule demonstrating the highest inhibitory action against the susceptible H37Rv MTB strain with MIC value of 4 µg/mL. Five Compounds (**2b**, **2d**, **3d**, **4a**, and **4b**) were found to be equipotent, with a MIC value of 8 µg/mL. Compounds **2c**, **3b**, and **4c** showed moderate anti-TB activity with a MIC value of 32 µg/mL against the same strain. Meanwhile, no activity was observed for the remaining indolizines against the H37Rv MTB strain.

**Table 2.** *In vitro* anti-mycobacterial activity of 1,2,3-trisubstituted indolizine derivatives (**2a–2f**, **3a–3d**, and **4a–4c**) against H37Rv and MDR strains of *Mycobacterium tuberculosis.* 
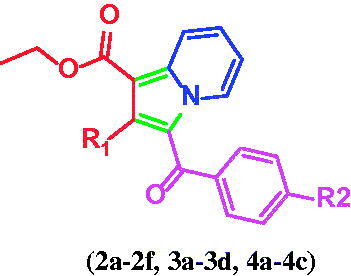

Entry	R_1_	R_2_	MIC (µg/mL)	
			H37Rv^a^	MDR-MTB^b^
**2a**	H	F	NA	NA
**2b**	H	Cl	8	NA
**2c**	H	Br	32	NA
**2d**	H	NO_2_	8	64
**2e**	H	CH_3_	NA	NA
**2f**	H	CN	NA	NA
**3a**	CH_3_	F	4	32
**3b**	CH_3_	Cl	32	NA
**3c**	CH_3_	Br	NA	NA
**3d**	CH_3_	NO_2_	8	NA
**4a**	CO_2_CH_2_CH_3_	F	8	16
**4b**	CO_2_CH_2_CH_3_	Cl	8	16
**4c**	CO_2_CH_2_CH_3_	NO_2_	32	64
**Rifampicin**			<1	≥1
**Isoniazid**			<0.2	≥0.2

^a^American Type Culture Collection (ATCC): 25177; ^b^these isolates were found to be resistant to the first line antibiotics, rifampicin (1 μg/mL), and isoniazid (0.2 μg/mL).

MDR-MTB: multidrug-resistant strains of *Mycobacterium tuberculosis*; MIC: minimum inhibitory concentration; NA: not active at the concentration range (0.2–64 μg/mL).

The structure–activity relationship (SAR) analysis revealed the presence of a hydrogen atom, a methyl or an ester substituent at the 2-position of the indolizine core was favourable to attain good anti-tubercular activity. The presence of the halogen and the nitro group at the *para* position of the benzoyl ring were found to be tolerated well to maintain the activity (except compounds **2a** and **3c**) against the H37Rv MTB strain, whilst the substitution with methyl and cyano group in compounds **2e** and **2f,** respectively, completely abolished the activity against both the MTB strains. In the case of nitro derivatives, compounds **2d** and **3d** with a hydrogen and methyl group, respectively, at the 2-position of the indolizine ring showed four-fold increased activity as compared with the compound **4c** with an ester group at the same position.

The structural alteration at the 2-position of the indolizine scaffold resulted in a profound impact on the anti-mycobacterial activity against MDR-MTB. The indolizine derivatives that were active (**2b–2d**, **3a**, **3b**, and **3d**) against the susceptible **H37Rv** MTB strain were found to be inactive against MDR-MTB, except for compounds **2d** and **3a** which exhibited 8-fold reduced MDR-MTB activity compared to H37Rv MTB strain (Table 2). However, compounds **4a-4c** with an ethyl ester group at the 4-position of the benzoyl ring showed moderate activity against MDR-TB, with a two-fold decrease in potency as compared with the cellular activity against susceptible H37Rv MTB strain. The anti-tubercular activity profile of the tested compounds against MDR-MTB provided a clue for their mode of action. Indeed, the results demonstrated that the first and second series of indolizines (**2a–2f** and **3b–3d**) did not show activity against MDR-MTB except for compounds **2d** and **3a**, while the third series of compounds **4a–4c** clearly inhibited the MDR-MTB strain. These findings suggest that the three designed series are likely to be inhibiting different MTB molecular drug targets; such that the first and second series of indolizine derivatives are inhibiting a common target that is different from that inhibited by the third series. Alternatively, the compounds that retained activity against MDR-MTB strain (third series) could be interacting with the same molecular target as those of the first two series, yet, might be interacting differently with the target binding site.

The above findings and observations supported the view that the first and second series of indolizine were more likely to inhibit the mycobacterial cell growth by interacting with the InhA enzyme. With regards to the mode of action of the ester indolizine series 3, their differences in inhibitory activity between susceptible strains and clinical isolates of MDR-MTB strains suggested that the derivatives **4a–4c** were interacting with another molecular target. Thus, further molecular modelling study was carried out to elucidate the potential mode of action of the tested compounds as well as to rationalise their SAR as inhibitors of InhA which represents one of the potential MTB therapeutic targets, and also to identify the second molecular target for the ester indolizine series 3.

### 3.5. Toxicity study

The promising activities of the designed compounds are very encouraging to undertake prospective optimisation cycles. However, it is highly recommended to optimise the ADMET properties for a lead compound early in the development stage to reduce later problems due to unfavourable ADMET characteristics^53^^,^^54^. Based on the *in silico* calculated ADME and toxicity parameters (Table 3), all the compounds are showing acceptable ADMET profile which makes them worthy of further optimisation towards achieving the desired drug-like properties.

**Table 3.** Calculated ADMET descriptors and toxicity parameters of indolizine derivatives (**2a–2f**, **3a–3d**, and **4a–4c**).

Compounds		MIC (µg/mL)		ADMET descriptors^a^								Toxicity parameters^b^								
Index	Code	H37Rv	MDR- MTB	AS	BBB	CYP2D6 inhibition	Hepatotoxicity	HIA	PPB	AlogP	PSA	AM	SI	OI	AB	DTP	CMR	CFR	CLM	CFM
1	2a	NA	NA	2	1	True	True	0	True	3.767	48.88	0	0.156	0.051	0	0	0.001	0	0.248	1
2	2b	8	NA	2	1	True	False	0	True	4.226	48.88	0	0.002	0.05	0	0	0.001	0	0	1
3	2c	32	NA	2	1	False	False	0	True	4.31	48.88	0	0	0.05	0.012	0	0.001	0	0	0.999
4	2d	8	64	2	3	False	True	0	True	3.456	91.703	0.062	0	0.84	0.001	0	0.008	0	0.976	1
5	2e	NA	NA	2	1	False	False	0	True	4.048	48.88	0	0	0.036	0	0	0	0	0	1
6	2f	NA	NA	2	2	False	False	0	True	3.441	71.815	0.945	0	0.359	0	0	0	0	0	1
7	3a	4	32	2	1	True	True	0	True	4.254	48.88	0	0.731	0.447	0	0.976	0.001	0	0.001	1
8	3b	32	NA	2	1	False	False	0	True	4.712	48.88	0	0.028	0.468	0	0.99	0.001	0	0	0.989
9	3c	NA	NA	2	1	False	False	0	True	4.796	48.88	0	0.006	0.476	0.985	0.992	0.001	0	0	0.861
10	3d	8	NA	2	2	False	True	0	True	3.942	91.703	0.012	0	0.984	0.129	0.956	0.008	0	0.188	0.996
11	4a	8	16	2	2	False	True	0	True	3.972	75.11	0	0.993	0.034	0.487	0.117	0.008	0	0	1
12	4b	8	16	2	1	False	False	0	true	4.43	75.11	0	0.478	0.04	0.521	0.239	0.008	0	0	0.599
13	4c	32	64	2	4	True	True	1	True	3.661	117.933	0.069	0.004	0.642	1	0.068	0.117	0	0.001	0.806

Aqueous solubility (AS)			Blood brain barrier (BBB) penetration			Human intestinal absorption (HIA)	
Level	Value	Drug-likeness	Level	Value	Description	Level	Description
0	log(Sw) <−8.0	Extremely low	0	Very High	Brain-Blood ratio greater than 5:1	0	Good absorption
1	−8.0 <log(Sw) <−6.0	No, very low, but possible	1	High	Brain-Blood ratio between 1:1 and 5:1	1	Moderate absorption
2	−6.0 <log(Sw) <−4.1	Yes, low	2	Medium	Brain-Blood ratio between 0.3:1 and 1:1	2	Low absorption
3	−4.1 <log(Sw) <−2.0	Yes, good	3	Low	Brain-Blood ratio less than 0.3:1	3	Very low absorption
4	−2.0 <log(Sw) ≤0.0	Yes, optimal	4	Undefined	Outside 99% confidence ellipse		
5	0.0 <log(Sw)	No, too soluble					

Probability values	Probability level	Description
0.0–0.30	Low probability	Such a chemical is not likely to produce a positive response in an experimental assay
Greater than 0.30 but less than 0.70	Intermediate probability	
Greater than 0.70	High probability	Likely to produce a positive response in an experimental assay

^a^Key to the above calculated ADMET descriptors.

^b^Key to the above calculated toxicity parameters.

Moreover, the toxicity profile for compounds **2d**, **3a**, **4a**, **4b**, and **4c** that showed promising activity against MDR-MTB was further assessed against peripheral blood mononuclear cells (PBMCs) and no signs of toxicity were observed with concentrations up to 256 µg/mL.

### 3.6. Computational molecular modelling

A molecular docking study was carried out to gain insight into the mechanism of action of the indolizine derivatives (**2a–2f**, **3a–3d**, and **4a–4c**). In light of the MTB inhibitory action, InhA appeared to be a potential molecular target for the title compounds; therefore these compounds were docked against InhA enzyme in an attempt to corroborate their mode of action with their observed anti-mycobacterial activity.

**Figure 7.**
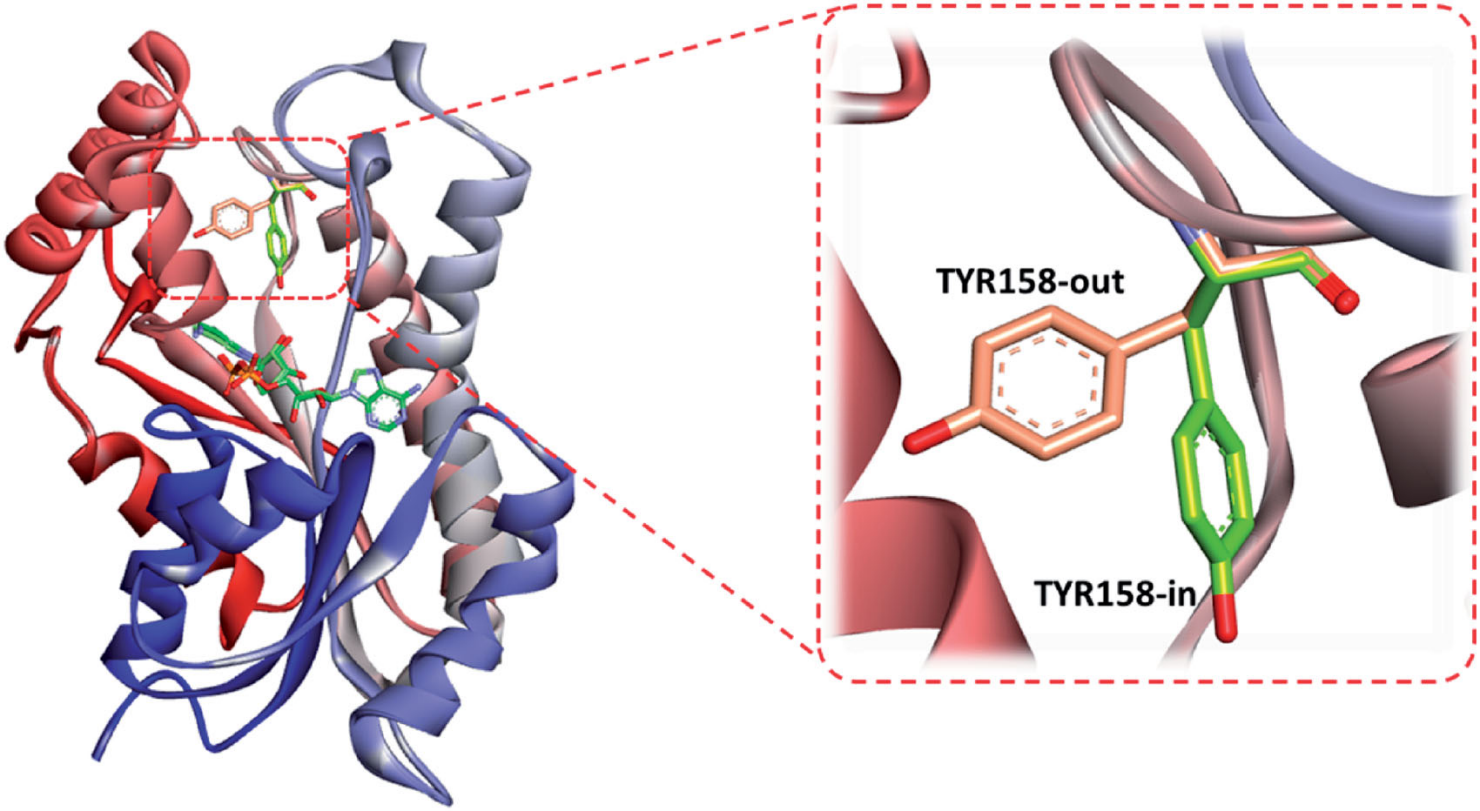
The two reported “in and out” conformations of Tyr158 in mycobacterial InhA enzyme. PDB ID for the in conformation is 5G0S, and that for the out conformation is 5G0U.

The binding feature common to all InhA inhibitors is the active participation of NAD, as a cofactor, contributing strong interaction with substrates through the H-bonding involvement from its ribose hydroxyl moiety. Additionally, the residue LYS 165 participates in the binding network by interacting with the hydroxyl oxygen of the NAD ribose through hydrogen bonding, and the residue ILE 91 is engaged in H-bonding with the amide functionality of the nicotinamide part of NAD. These residues greatly contributed to the stability of the cofactor. Hence, the latter tends to play a critical role in molecular interaction with the substrate and other residues in the InhA binding domain. Consequently, the presence of NAD during a docking study may have a profound effect not only on the ligand interaction but also on the occupancy size of the active site that may affect ligand orientation. Besides, a great majority of reported InhA inhibitors make a stable hydrogen bonding to the backbone hydroxyl group of the main chain TYR 158, responsible for blocking the enoyl-acyl carrier protein reductase function^55^. The role of TYR 158 in the catalytic mechanism of InhA has been the focus of many studies which revealed that its side chain can adopt different conformations when direct InhA inhibitors bind the catalytic site. Mainly two conformations were identified, an “in” and “out” conformations. The former is resembling the substrate-NAD-InhA ternary complex, and the latter resembles the NAD-InhA binary complex (Figure 7); however, in the majority of available InhA-inhibitor complexes TYR158 is in the “in” conformation and the inhibitor is forming interactions with the cofactor^56–58^. However, a novel binding mode of a benzimidazole-based InhA inhibitor has been recently disclosed, occupying an extended hydrophobic pocket formed by the amino acid residues Phe149, Met155, Pro156, Ala157, and Ile215 in the in-Tyr 128 conformation^57^. Certain InhA inhibitors were also reported to co-crystallize with Tyr 158-out active site residue^59–65^.

**Figure 8.**
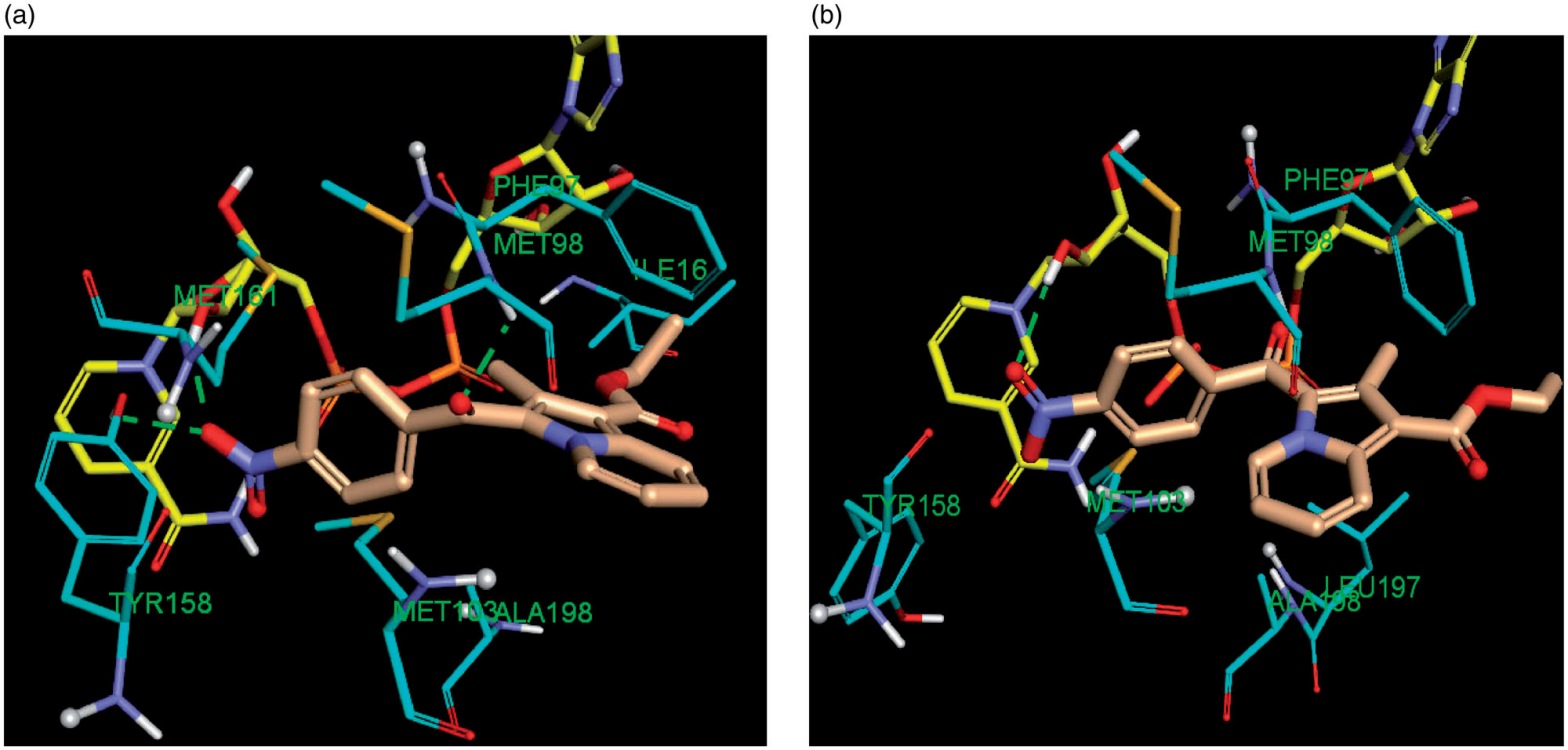
Comparative binding mode of indolizine **2d** into InhA binding domain with (a) in-Tyr 158 (PDB ID: 5G0S) and (b) out-Tyr 158 conformations (PDB ID: 5G0U), respectively. The ligand, NAD and the receptor were represented as sticks in solmon, yellow and cyan colours respectively. The hydrogen bonds are shown as green dotted lines.

Accordingly, the binding mode of the indolizine **2d** was investigated by docking it into the active site of the InhA enzyme utilising crystal structures of the in and out conformations, namely 5G0S and 5G0U respectively Figure 8.

**Figure 9.**
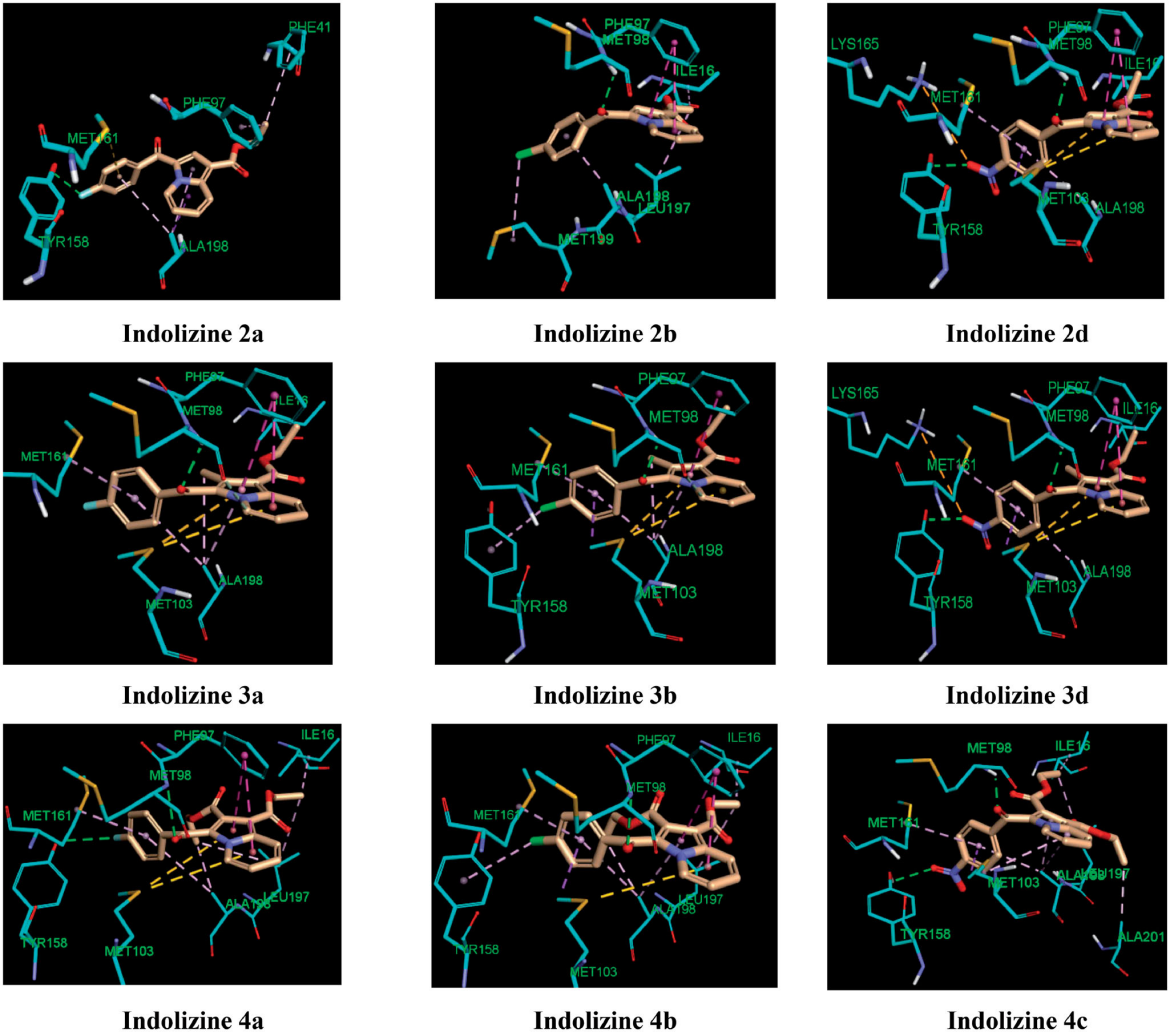
Predicted binding interaction of indolizines (**2a**, **2b**, **2d**, **3a**, **3b**, **3d**, **4a–4c**) with InhA binding domain (PDB 5G0S). Ligand and receptor were represented as solmon and cyan respectively. Hydrogen bonding contact is shown with green dotted lines. π–π, π–sulphur and hydrophobic interactions are shown with magenta, gold and violet, respectively. For displaying key bingind interaction, NAD has been omitted.

The docking results revealed that the estimated binding energy of **2d** was −107.46 kcal/mol and −144.02 kcal/mol with the InhA binding site 5G0U (Tyr 158-out) and 5G0S (Tyr 158-in) respectively, indicating that the indolize **2d** interacted more favourably with the in Tyr 158 conformation active residue. Indeed, the binding pose of **2d** showed three hydrogen bond contacts with active site’s residues in the “in” conformation (pdb 5G0S), while only one with the “out” conformation (pdb 5G0U) (Figure 8). The binding mode of **2d** revealed that the benzoyl ring occupies the same location in both the crystal involving H-bond interaction with the co-factor NAD but differ on the orientation of the indolizine ring (Figure 8). In light of the comparative docking analysis the InhA crystal structure of 5G0S, “in” conformation, appeared as more appropriate to investigate the binding patterns. Hence, docking of the remaining derivatives was performed with the Tyr 158-in conformation InhA active site (PDB 5G0S). The docking results, reported in Table 4, indicated that the active indolizines **2b**–**2d**, **3a**, **3b,** and **4a**–**4c** displayed greater binding energy as compared to that of inactive congeners **2a**, **2e**, **2f**, and **3c**. In general, the predicted docking energy of indolizines was consistent with the observed MTB inhibitory activity of the tested compounds, except for the compounds **2d**, **3d**, and **4c** with a nitro substitution (Figure 9). However, the order of the potency in the second indolizine series was **3a** (F) > **3b** (Cl) ≫ **3c** (Br) which correlates with the docking scores. A similar correlation between the activity and docking scores was also observed in the first indolizine series in which the order of bioactivity was **2b** (Cl) > **2c** (Br) ≫ **2a** (F), **2f** (CN), **2e** (CH_3_). Among the compounds **2a** and **3a**, the most active compound **3a** (F) displayed a higher binding energy score than the inactive compound **2a** (F). The compound **2b** (Cl) being more active than compound **3b** (Cl) demonstrated a higher energy score. A similar trend was also observed for the bromine derivatives **2c** and **3c**. The nitro compounds **2d** and **3d** were equipotent and showed comparable docking scores. As for the third series, the indolizines **4a**–**4c** showed very high docking scores. The equipotent indolizines **4a** (F) and **4b** (Cl) showed comparable docking scores, whilst the nitro derivative **4c** showing a higher docking score was four-fold less potent than the compounds **4a** and 4**b**.

**Table 4.** Docking results of the indolizine derivatives **2a**–**2f**, **3a–3d** and **4a**–**4c** into the InhA binding domain (PDB 5G0S).

Entry	R_1_	R_2_	Binding E. (Kcal/mol)	Residues Interaction	
				H-Bond (Dist. Å, atom)	Pi-interaction
**2a**	H	F	−114.75	Tyr 158 ( 2.71, F)	
**2b**	H	Cl	−128.17	Met 98 (2.32, CO benzoyl)	Phe 97 (Pi–Pi)
**2c**	H	Br	−120.18	Met 98 (2.37, CO benzoyl)	Phe 97 (Pi–Pi)
**2d**	H	NO_2_	−144.02	Met 98 (2.13, CO benzoyl) Tyr 158 (2.56, NO_2_) NAD (2.00, NO_2_)	Phe 97 (Pi–Pi) Met 103 (Pi–S)
**2e**	H	CH_3_	−97.47		
**2f**	H	CN	−99.48		
**3a**	CH_3_	F	−152.37	Met 98 (2.27, CO benzoyl) NAD (2.19, F)	Phe 97 (Pi–Pi) Met 103 (Pi–S)
**3b**	CH_3_	Cl	−124.20	Met 98 (2.15, CO benzoyl)	Phe 97 (Pi–Pi) Met 103 (Pi–S)
**3c**	CH_3_	Br	−104.21		
**3d**	CH_3_	NO_2_	−147.40	Met 98 (2.13, CO benzoyl) Tyr 158 (2.55, NO_2_) NAD (1.99, NO_2_)	Phe 97 (Pi–Pi) Met 103 (Pi–S)
**4a**	CO_2_CH_2_CH_3_	F	−165.58	Met 98 (2.32, CO benzoyl) Tyr 158 (3.53, F) NAD (2.82, F)	Phe 97 (Pi–Pi) Met 103 (Pi–S)
**4b**	CO_2_CH_2_CH_3_	Cl	−165.95	Met 98 (2.45, CO benzoyl)	Phe 97 (Pi–Pi) Met 103 (Pi–S)
**4c**	CO_2_CH_2_CH_3_	NO_2_	−182.03	Met 98 (2.04, CO benzoyl) Tyr 158 (2.93, NO_2_) NAD (2.01, NO_2_)	
Native ligand			−186.54	Met 98 (1.97) Gln 100 (2.41) Tyr 158 (2.82) NAD (2.06 )	Phe 149 (Pi − Pi)

**Figure 10.**
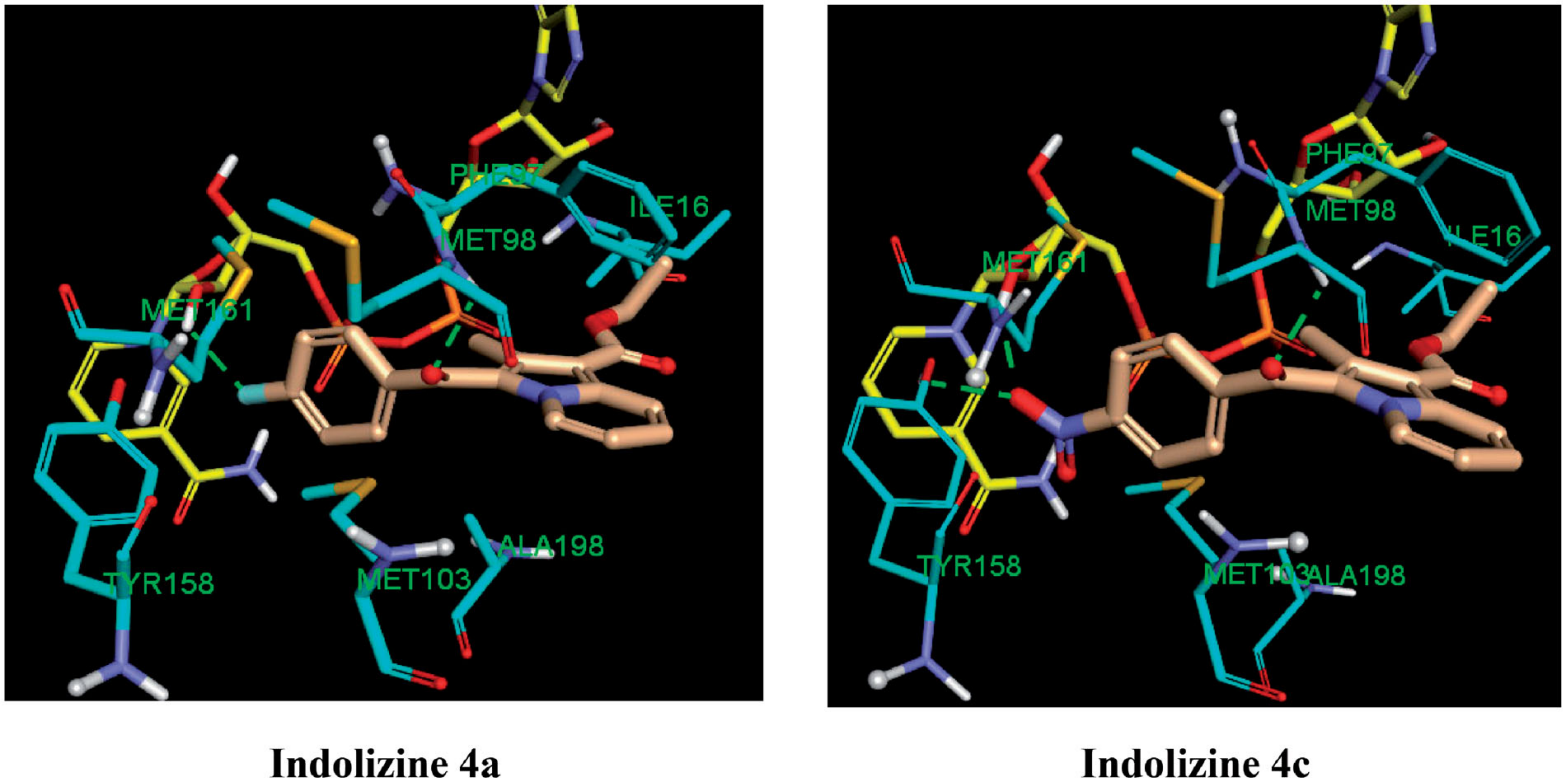
Predicted binding interaction of indolizines (**4a**, **4c**) with InhA binding domain (PDB 5G0S). Ligand, NAD and receptor were represented as solmon, yellow and cyan respectively. Hydrogen bonding contact is shown with green dotted lines.

The observed MTB inhibitory activity pattern of the indolizines can be accounted for their distinctive feature of binding mode into the InhA active site. The key binding interaction of the title compounds was highlighted in Figures 9 and 10. The binding poses of the title compounds showed that the benzoyl ring is located near to the co-factor binding site with a sandwich orientation between the nicotinamide ring of NAD and the main chain Tyr 158 which is consistent with the binding mode of known InhA inhibitors. The active indolizines **2b**–**2d**, **3a**, **3b**, and **4a**–**4c** were predicted to adopt a similar conformation in the binding domain by forming strong hydrogen bonding with the residue Met 98 and the carbonyl moiety of the benzoyl group, whereas the carbonyl moiety of the benzoyl group for the inactive indolizines **2a**, **2e**, **2f**, and **3c** was orientated away from the residue Met 98 preventing the H-bonding interaction (Figure 9). The conformation alteration from the rotation of the benzoyl group could provide a rationale for the lack of potency observed for these derivatives. However, the interaction of the residue Met 98 with the native ligand and certain InhA inhibitors is reported as well^61^^,^^62^. It was also demonstrated that interacting with the Met 98 residue through H-bonding was the key interaction to retain the MTB inhibitory activity when the main chain Tyr 158 was not involved in any hydrogen bonding with the ligand.^59^ Derivatives **2d**, **3a**, **3d**, **4a**, and **4c** were able to make additional hydrogen bonding contacts with residues in the active site. For instance, the *para* nitrobenzoyl indolizines **2d**, **3d**, and **4c** showed two H-bonding interactions between the nitro group and the residue Tyr 158 and the hydrogen atom of the hydroxyl group from the NAD ribose (Figure 10). Similarly, the derivative **4a** having a fluorine atom in the para position of the benzoyl moiety showed a moderate hydrogen bonding contact with the hydroxyl group of the NAD ribose and a weak H-bond with the residue Tyr 158, while compound **3a** demonstrated only one strong H-bond with the hydroxyl of the NAD ribose but exempting from any H-bond interaction with the residue Tyr 158 (Figure 10). The strength of H-bonding involvement with NAD ribose may explain the greater potency observed for the derivative **3a** (2.19 Å) as compared to the compound **4a** (2.82 Å).

Another important contribution to the cellular activity of the bioactive compounds is the hydrophobic interaction implicated by the residues Phe 97 and Met 103 (Figure 9 and Table 4), Where Phe 97 formed T-shaped pi-stacking interaction with the indolizine ring, except for compound **4c**, and the residue Met 103 was observed to interact with the indolizine ring through pi-sulfur interactions with the exception of compounds **2b** and **4c**. The lack of hydrophobic interaction with the residues Phe 97 and Met 103 for the compound **4c** (NO_2_) provides an explanation for its reduced potency in comparison with its nitro congeners **2d** and **3d** (Figure 9).

The molecular modelling analysis indicated that the observed MTB potency of indolizines was in good agreement with the predicted binding mode and binding energy predictions. These computational results strongly support our assumption that the molecular target of the designed indolizine is enoyl-acyl carrier protein reductase, and the key interactions with the residues Met 98 and Phe 91 were highlighted as being mainly responsible for the observed activity of the compounds. These findings could provide a valuable guide to designing more potent indolizines as potential InhA inhibitors through a structure-based design approach.

**Figure 11.**
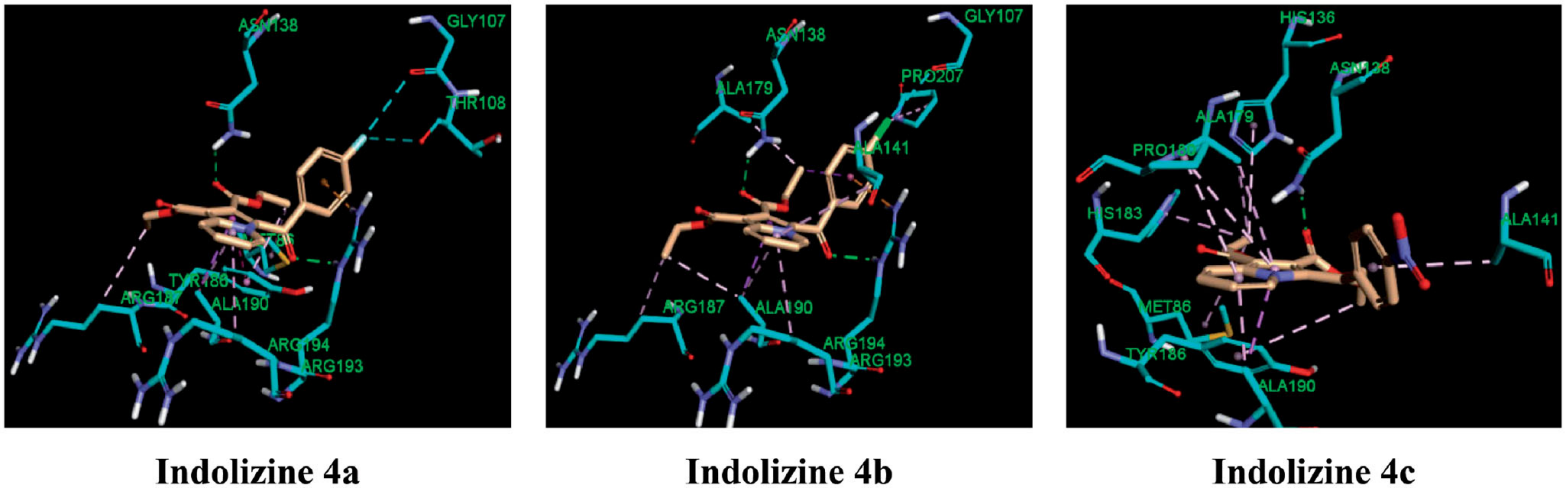
Predicted binding interaction of indolizines (**4a–4d**) with anthranilate phosphoribosyl binding domain (PDB 3R6C). Ligand and receptor were represented as solmon and cyan respectively. Hydrogen bonding contact is shown with green dotted lines. Halogen bonding and hydrophobic intreactions are shown with cyan and violet respectively.

We next focussed on the prospective identification of the cellular target of the ester derivatives **4a**–4**c**. According to the cellular activity profile against MDR MTB, the ester indolizine series **4a–4c**, identified as potential InhA inhibitors, may have more than one molecular MTB target. In the quest to identify the second potential target for the indolizines **4a**–**4c**, again, computational molecular docking studies were carried out. Based on the MTB activity of derivatives **4a**–4**c** against both susceptible and resistant MTB strains, we hypothesised that the presence of the ester functional group at 2-position of the indolizine may strongly contribute to the bioactivity through the formation of hydrogen bonding contact. Furthermore, it has been observed that the indolizine **4a** and **4b**, having halogen substituent in the para position of the benzoyl moiety, had exhibited identical anti-TB activity and were more active than the nitro derivative **4c**, indicative of probable involvement of halogen bonding interaction with the receptor. With regards to the above considerations, a large number of known TB molecular targets were screened. The molecular target anthranilate phosphoribosyltransferase appeared to be the appropriate enzyme target for indolizines **4a**–4**c**. The docking scores, presented in Table 5, were also found to be consistent with the MTB activity of the compounds in which the equipotent indolizines **4a** and **4b** displayed similar binding energy, while the less active indolizine **4c** showed a lower docking score. The binding interaction of the indolizines **4a**–4**c** with the anthranilate phosphoribosyl enzyme was illustrated in Figure 11. The most active compounds **4a** and **4b** were found to adopt similar orientation in the anthranilate phosphoribosyl binding domain showing two strong hydrogen bonding contacts between the carbonyl group of benzoyl and ester at 2-position of indolizine ring with the amino acid residues Arg 193 and Asn 138, respectively. Among the numerous hydrophobic interactions, the fluorine atom of compound **4a** showed halogen interaction with the residues Gly 107 and Thr 108, similarly, the chlorine atom of compound **4b** exhibited interaction with the residue Gly 107. Both indolizines **4a** and **4b** were also involved in pi-cation interaction between the benzoyl ring of the indolizine and the residue Arg 193. The binding mode of the indolizine **4c** differed from its congeners demonstrating only one H-bond contact between the residue Asn 138 and the carbonyl of ester group at the 2-position of indolizine ring, explaining the lower observed MTB activity. However, although the above computational results reasonably explained the observed anti-tubercular activities of the tested compounds, yet, they need to be experimentally verified and validated via biomolecular *in vitro* testing. Thus, it could be the subject of prospective research due to its importance in confirming the *in silico* results that is imperative to guide prospective optimisation of these lead compounds towards designing more potent and selective drug candidates.

**Table 5.** Docking results of the indolizine derivatives **4a**–**4c** into the anthranilate phosphoribosyl binding domain (PDB 3R6C).

Entry	R_1_	R_2_	Binding E. (Kcal/mol)	Residues Interaction	
				H-Bond (Dist. Å, atom)	Hydrophobic
**4a**	CO_2_Et	F	−170.37	Arg 193 (2.09, CO benzoyl) Ans 138 (2.41, CO ester)	Gly 107 (3.58, F) Thr 108 (3.51, F) Arg 193 (3.40, Pi-cation)
**4b**	CO_2_Et	Cl	−166.81	Arg 193 (2.16, CO benzoyl) Ans 138 (2.24, CO ester)	Gly 107 (3.22, Cl) Arg 193 (3.31, Pi-cation)
**4c**	CO_2_Et	NO_2_	−140.06	Ans 138 (2.15, CO ester)	

## 4. Conclusion

In the current study, three novel series of 1,2,3-trisubstituted indolizine derivatives (**2a–2f**, **3a–3d**, and **4a–4c**) were evaluated for their anti-TB activity against susceptible and MDR-MTB strains. The majority of tested compounds showed good to excellent anti-tubercular activity with MIC ranging from 4 to 64 µg/mL. The inhibition profile of indolizines and molecular docking strongly supported that InhA is the principal drug target for the investigated compounds. Molecular modelling insight indicated that the conformation changes resulting from the benzoyl group rotation provided a rationale for the MTB cellular activity of the indolizines as InhA inhibitor. Furthermore, the cellular activity profile of the ester indolizine derivatives (**4a-4c**) against both the strains revealed their inhibitory action in multiple molecular targets in which anthranilate phosphoribosyltransferase might be a potential additional drug target. Our results highlighted the importance of indolizines as a novel promising class of multi-targeting agents for MTB with favourable toxicity profile. Such compounds might serve as attractive leads for further development of potential drug candidates for combating both the drug-sensitive and drug-resistant tuberculosis strains.

The compound **4b** was crystallised in a triclinic centrosymmetric crystal system with space group *P-1*. In stabilising the molecular structure, the C–H···O and other short contact have played an important role. Hirschfeld surface analysis with 2D fingerprint plots was performed to provide insight into the stability of the crystal structure in order to understand and visualise the contribution of distinct intermolecular interactions. Systematic and theoretical energy was measured in terms of electrostatic, dispersion, and total energy using the program *Crystal Explorer* software, which further depicts 3D topological images.

## Supplementary Material

Supplemental Material
