# Supplementary material for: Anti-tubercular activity and molecular docking studies of indolizine derivatives targeting mycobacterial InhA enzyme
Source: J Enzyme Inhib Med Chem. 2021 Jul 1;36(1):1472–87. doi: 10.1080/14756366.2021.1919889 (PMC8259857; doi:10.1080/14756366.2021.1919889)
Supplement: Supplemental Material [file IENZ_A_1919889_SM0236.pdf]

Anti-Tubercular Activity and Molecular Docking Studies of Indolizine Derivatives

Katharigatta N. Venugopala[1,2,*], Sandeep Chandrashekharappa[3], Pran Kishore Deb[4*], Christophe Tratrat[1], Melendhran Pillay[5], Deepak Chopra[6], Nizar A. Al-Shar'i[7], Wafa Hourani[4], Lina A. Dahabiyeh[8], Pobitra Borah[9], Rahul D. Nagdeve[10], Susanta K. Nayak[10], Basavaraj Padmashali[11], Mohamed A. Morsy[1,12], Bandar E. Aldhubiab[1], Mahesh Attimarad[1], Anroop B. Nair[1], Nagaraja Sreeharsha[1,13], Michelyne Haroun[1], Sheena Shashikanth[14], Viresh Mohanlall[2] Raghuprasad Mailavaram[15]

[1] Department of Pharmaceutical Sciences, College of Clinical Pharmacy, King Faisal University, Al-Ahsa 31982, Saudi Arabia;
[2] Department of Biotechnology and Food Technology, Durban University of Technology, Durban 4001, South Africa;
[3] Institute for Stem Cell Science and Regenerative Medicine (inStem), UAS-GKVK Campus, Bellary Road, Bangalore, Karnataka 560065, India;
[4] Department of Pharmaceutical Sciences, Faculty of Pharmacy, Philadelphia University, Amman 19392, Jordan;
[5] Department of Microbiology, National Health Laboratory Services, KZN Academic Complex, Inkosi Albert Luthuli Central Hospital, Durban 4001, South Africa;
[6] Department of Chemistry, Indian Institute of Science Education and Research Bhopal, Bhopal By-pass Road, Bhauri, Bhopal 462066, Madhya Pradesh, India;
[7] Department of Medicinal Chemistry and Pharmacognosy, Faculty of Pharmacy, Jordan University of Science and Technology, P.O. Box 3030, Irbid, 22110, Jordan;
[8] Department of Pharmaceutical Sciences, School of Pharmacy, The University of Jordan, Amman, Jordan;
[9] Pratiksha Institute of Pharmaceutical Sciences, Chandrapur Road, Panikhaiti, Guwahati 781026, Assam, India;
[10] Department of Chemistry, Visvesvaraya National Institute of Technology, Nagpur 440010, Maharashtra, India;
[11] Department of Chemistry, School of Basic Science, Rani Channamma University, Belagavi 591156, India;
[12] Department of Pharmacology, Faculty of Medicine, Minia University, El-Minia 61511, Egypt;
[13] Department of Pharmaceutics, Vidya Siri College of Pharmacy, Off Sarjapura Road, Bangalore 560035, India;
[14] Department of Studies in Organic Chemistry, University of Mysore, Manasagangotri, Mysore 570006, India;
[15] Pharmaceutical Chemistry Division, Sri Vishnu College of Pharmacy, Bhimavaram (534202), West Godavari, Andhra Pradesh, India

*** Corresponding authors:**

Dr. Katharigatta N. Venugopala
Associate Professor,
Department of Pharmaceutical Sciences, College of Clinical Pharmacy, King Faisal University, Al-Ahsa 31982, Saudi Arabia
Email: kvenugopala@kfu.edu.sa;
Tel.:+966-1358-98842

Dr. Pran Kishore Deb
Associate Professor
Department of Pharmaceutical Sciences, Faculty of Pharmacy, Philadelphia University, Amman 19392, Jordan
Email: prankishore1@gmail.com;
Tel.: +962-77720811

TABLE OF CONTENTS

S. No	Description	Page numbers
1.	**Energy framework calculation**	3
2.	**Table S1.** Single crystal X-ray data of title compound diethyl 3-(4-chlorobenzoyl)indolizine-1,2-dicarboxylate (**4b**).	4
3.	**Table S2.** Interaction Energies as obtained from the *Crystal Explorer 17.5* (in KJ/mol).	5
4.	**Figure S1**: FT-IR of diethyl-3-(4-fluorobenzoyl)indolizine-1,2-dicarboxylate (**4a**)	6
5.	**Figure S2**: ^{1}H-NMR of diethyl-3-(4-fluorobenzoyl)indolizine-1,2-dicarboxylate (**4a**)	7
6.	**Figure S3**: ^{13}C-NMR of diethyl-3-(4-fluorobenzoyl)indolizine-1,2-dicarboxylate (**4a**)	8
7.	**Figure S4**: FT-IR of diethyl-3-(4-chlorobenzoyl)indolizine-1,2-dicarboxylate (**4b**)	9
8.	**Figure S5**: ^{1}H-NMR of diethyl-3-(4-chlorobenzoyl)indolizine-1,2-dicarboxylate (**4b**)	10
9.	**Figure S6**: ^{13}C-NMR of diethyl-3-(4-chlorobenzoyl)indolizine-1,2-dicarboxylate (**4b**)	11
10.	**Figure S7**: FT-IR of diethyl-3-(4-nitrobenzoyl)indolizine-1,2-dicarboxylate (**4c**)	12
11.	**Figure S8**: ^{1}H-NMR of diethyl-3-(4-nitrobenzoyl)indolizine-1,2-dicarboxylate (**4c**)	13
12.	**Figure S9:** ^{13}C-NMR of diethyl-3-(4-nitrobenzoyl)indolizine-1,2-dicarboxylate (**4c**)	14
13.	**Figure S10.** Energy frameworks corresponding to the total interaction energy between the selected molecule **4b** and the molecules present in a 3.8 A° cluster around it.	15
14.	**Figure S11.** d_{norm} mapped on Hirshfeld surface of the molecule **4b** with energy framework in the form of **(a)** Coulombic energy, **(b)** dispersion energy and **(c)** total energy.	15
15.	**Figure S12.** Crystal voids corresponds to promolecule surface including all the atoms in the title compound **4b.**	16
16.	**Figure S13.** Crystal voids present in the title compound **4b** along with ***ac*** plane, ***bc*** plane and ***ab*** plane respectively.	16
17.	**References**	17

1. Energy framework calculation

The software *Crystal Explorer 17.5* program has been used to assess the interaction energies for diethyl 3-(4-chlorobenzoyl)indolizine-1,2-dicarboxylate (**4b**). The supramolecular nature of molecular crystal structures has an intense and unusual way of imagining energy frameworks. At the B3LYP/6-31G(d,p) level, the interaction energies between the molecules are obtained using monomer wave functions. In all the energy frameworks, the tube size (scale factor) used was 80, and the energy threshold (cut off) value was set to zero. For the corresponding interaction, the diameter of the tube cylinder represents the interaction energy in the molecular packaging in the 3D-topological images. Interaction between the molecule selected and the molecules present in the 1x1x1 unit cell dimensions of a 3.8 A° cluster around it, as shown in **Figure S10**. Energies between molecular pairs are described as cylinders which connect centroids of molecular pairs with a cylindrical radius proportional to the magnitude of the energy interaction. The energy framework was modelled as red cylinders for E_{elec}, E_{dis} as green, and E_{tot} as blue [**Figure S11a-S11c**], and the relative strength of molecular packing in various directions is expressed by these tubes. Therefore, energy structures precisely imagine the supramolecular nature of the crystal structure. Interaction Energies as obtained colourwise from the *Crystal Explorer 17.5* software in the form of KJ/mol as shown in **Table S1**. As shown in **Figure S12**, the crystal void generates a promolecule surface, including all the atoms in the cluster present in the crystal packing. The void surface is known as an isosurface of procrystal electron density in *Crystal Explorer* program and calculated for a whole unit cell [1-3]. The default value is 0.002 a.u. The void volume in **Figure S12** is 104.53 Å^3, and the surface area is 309.58 Å^2. The observed value of void volume for compound **4b** in **Figure S12** shows that there are no large cavities found in the anhydrous form. Crystal voids present in the title compound **4b** along with ***ac*** plane, ***bc*** plane and ***ab*** plane respectively as shown in the **Figure S13**.

Table S1. Single crystal X-ray data of title compound diethyl 3-(4-chlorobenzoyl)indolizine-1,2-dicarboxylate (**4b**).

Parameter	Value
CCDC Number	2002636
Molecular Formula	$C_{21}H_{18}C1_{1}N_{1}O_{5}$
Molecular weight	399.81
Temperature	100(2)
Crystal Size (mm)	0.15, 0.15, 0.14
Absorption coefficient (mm^{-1})	0.238
T_{min}, T_{max}	0.966,1.000
Crystal system	Triclinic
Lattice parameters: a (Å), b (Å), c (Å)	8.6556(4), 10.1462(5), 11.8762(6)
α, β, γ(°)	71.173(2), 73.631(2), 76.169(2)
Space Group, Density, Z, Z′	*P-1*, 2, 1
$h_{min, max}$; $k_{min, max}$; $l_{min, max}$;	-11,11; -13, 13; -15, 15
Number of total/unique/observed reflections	23691,4643, 3311
No of parameters	255
R_{int}	0.0608
R_{all}, R_{obs}	0.0788, 0.0454
$wR2_{all}$, $wR2_{obs}$	0.1043, 0.0879
$\Delta\rho_{min,max}$ ($eÅ^{-3}$)	-0.390, 0.317
G.o.F	1.050

Table S2. Interaction energies as obtained from the *Crystal Explorer 17.5* (in KJ/mol).

Colour	N	Symop	R	E_ele	E_pol	E_dis	E_rep	E_tot
Red	2	x, y, z	8.66	0.1	-0.2	-11.5	3.6	-7.8
Orange	1	-x, -y, -z	5.94	-28.3	-10.1	-58.6	51.8	-56.4
Yellow	1	-x, -y, -z	7.58	-9.8	-1.5	-59.4	32.2	-43.3
Lime	1	-x, -y, -z	15.68	0.6	-0.1	-4.2	1.3	-2.3
Green	2	x, y, z	11.88	-0.1	-0.1	-5.6	1.3	-4.3
Aquamarine	1	-x, -y, -z	6.67	-19.0	-5.7	-118.5	70.3	-84.2
Cyan	2	x, y, z	10.15	-13.4	-3.1	-24.0	21.3	-24.2
Blue	1	-x, -y, -z	11.66	-8.3	-1.0	-25.1	26.0	-15.3
Violet	1	-x, -y, -z	8.97	-12.3	-3.5	-15.0	16.3	-18.6
Orchid	2	x, y, z	12.57	-3.4	-1.2	-14.7	8.8	-11.9
Pink	1	-x, -y, -z	8.02	-19.3	-9.1	-27.4	19.6	-38.9

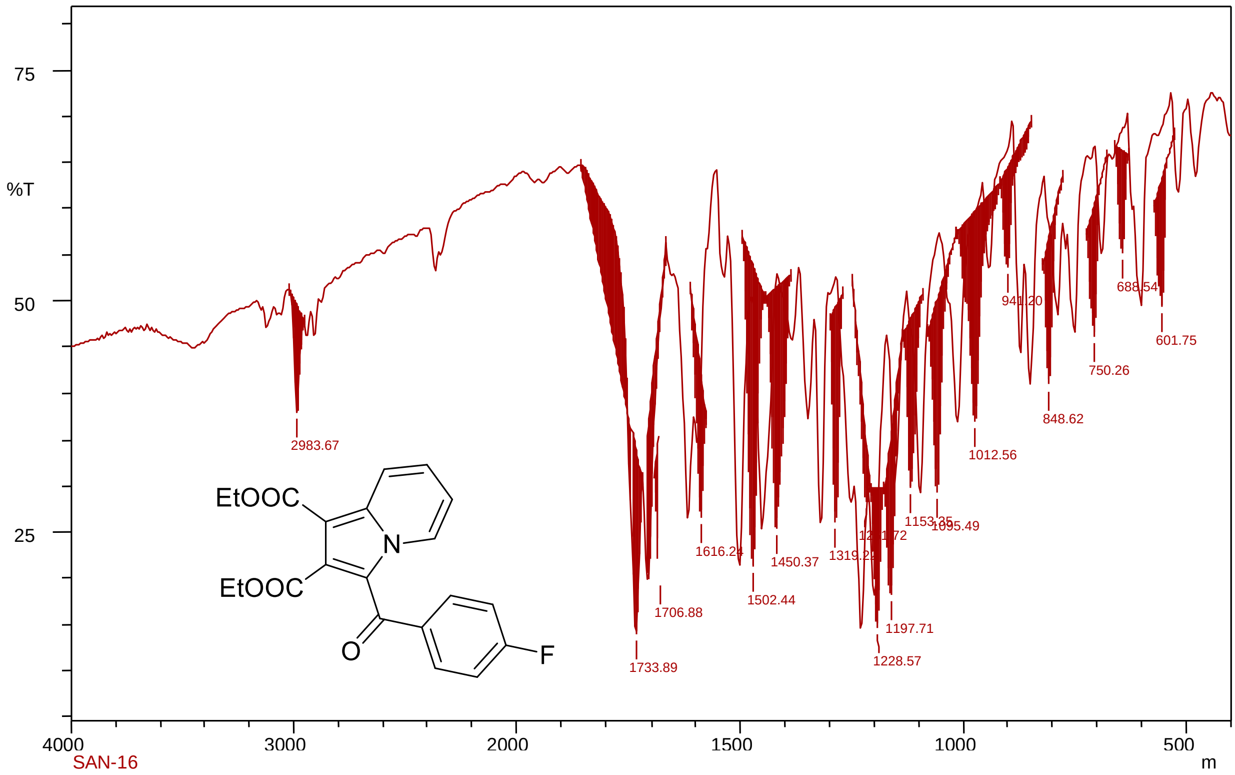

Figure S1: FT-IR of diethyl-3-(4-fluorobenzoyl)indolizine-1,2-dicarboxylate (**4a**)

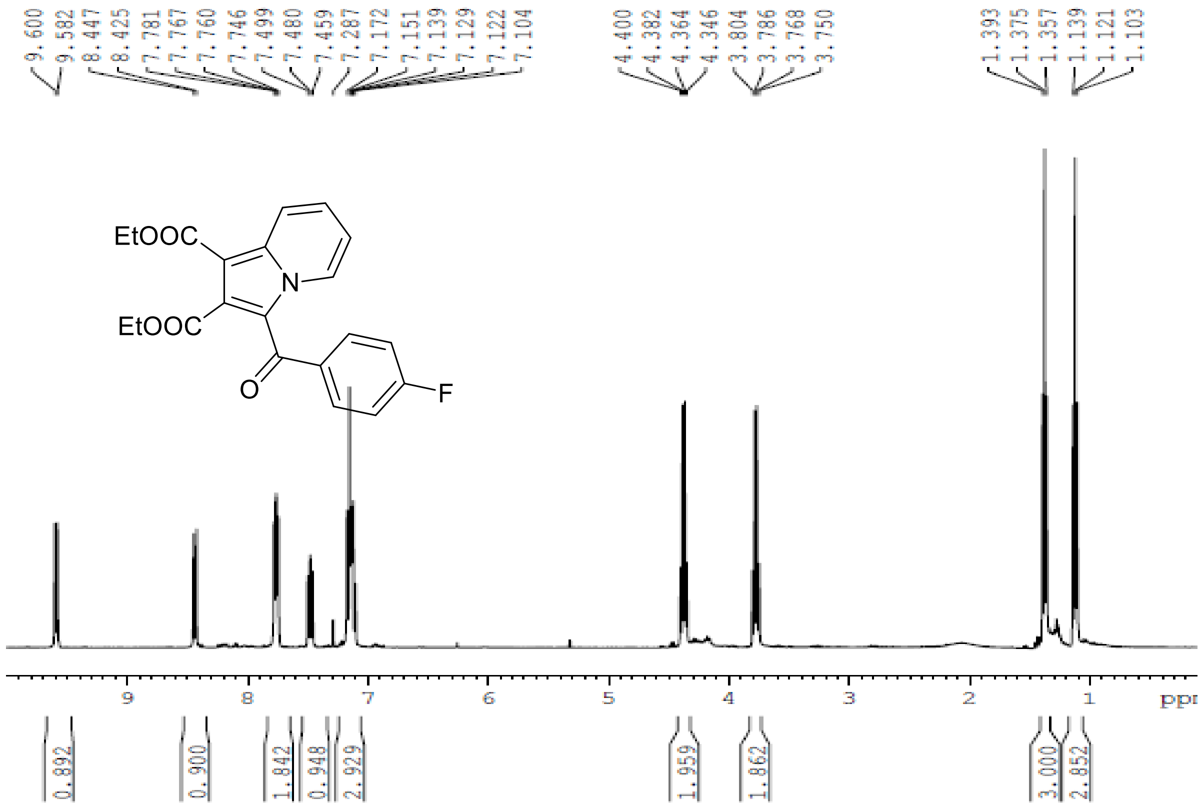

Figure S2: ^{1}H-NMR of diethyl-3-(4-fluorobenzoyl)indolizine-1,2-dicarboxylate (**4a**)

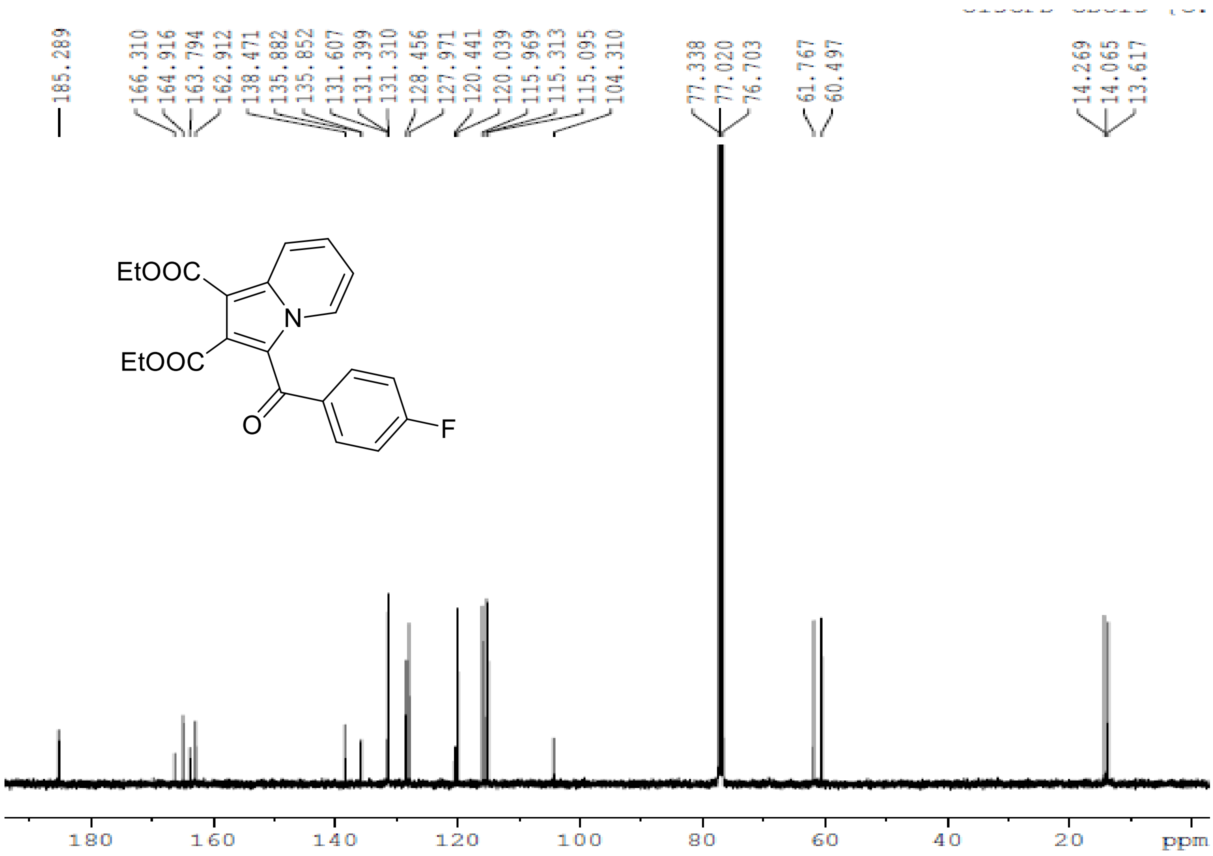

Figure S3: ^{13}C-NMR of diethyl-3-(4-fluorobenzoyl)indolizine-1,2-dicarboxylate (**4a**)

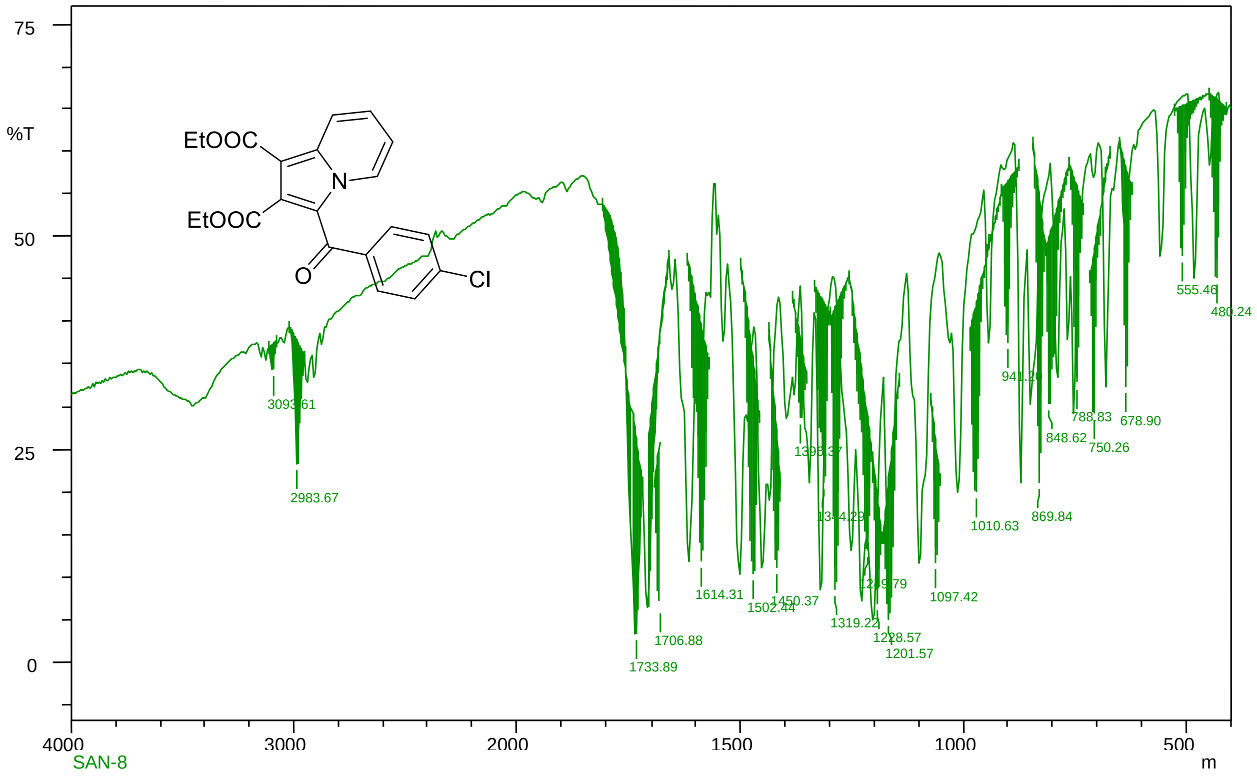

Figure S4: FT-IR of diethyl-3-(4-chlorobenzoyl)indolizine-1,2-dicarboxylate (**4b**)

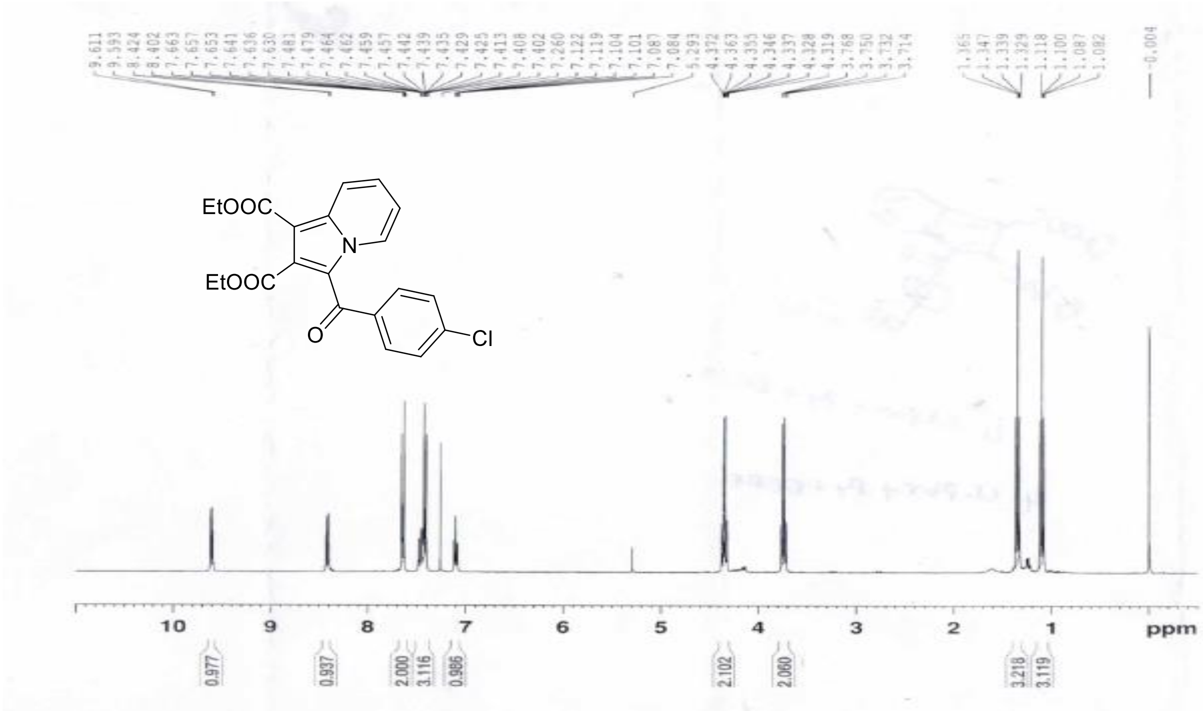

Figure S5: ^{1}H-NMR of diethyl-3-(4-chlorobenzoyl)indolizine-1,2-dicarboxylate (**4b**)

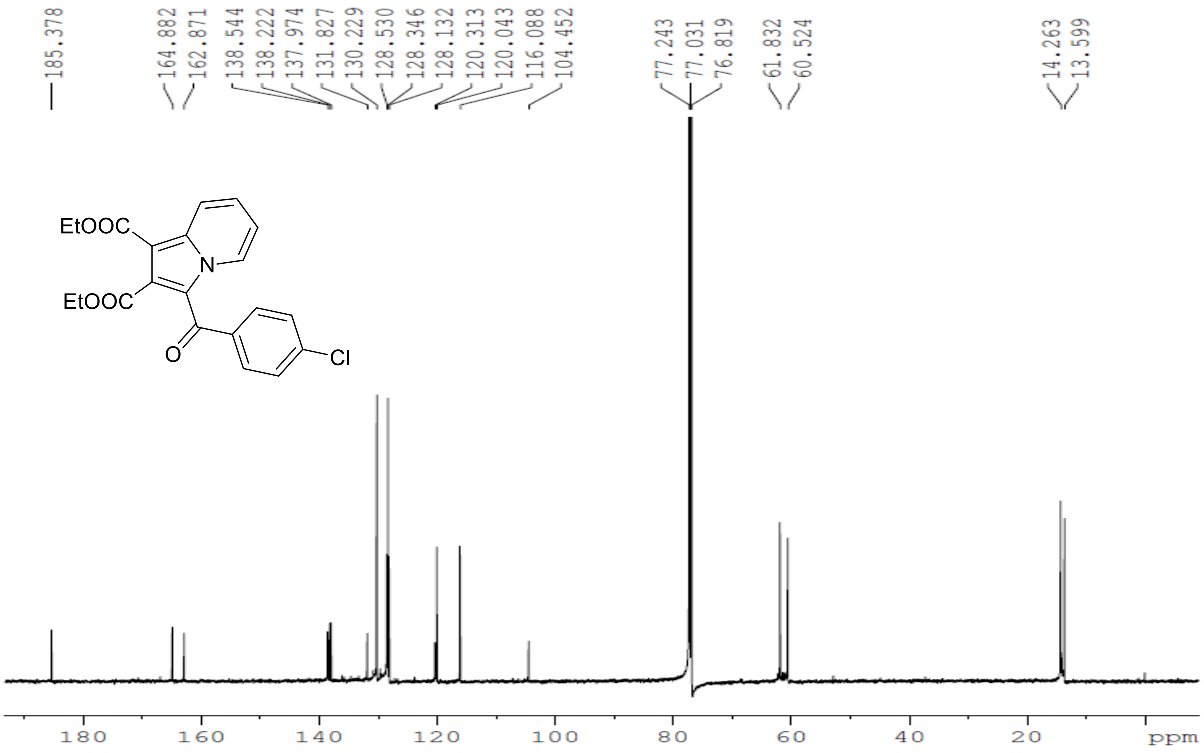

Figure S6: ^{13}C-NMR of diethyl-3-(4-chlorobenzoyl)indolizine-1,2-dicarboxylate (**4b**)

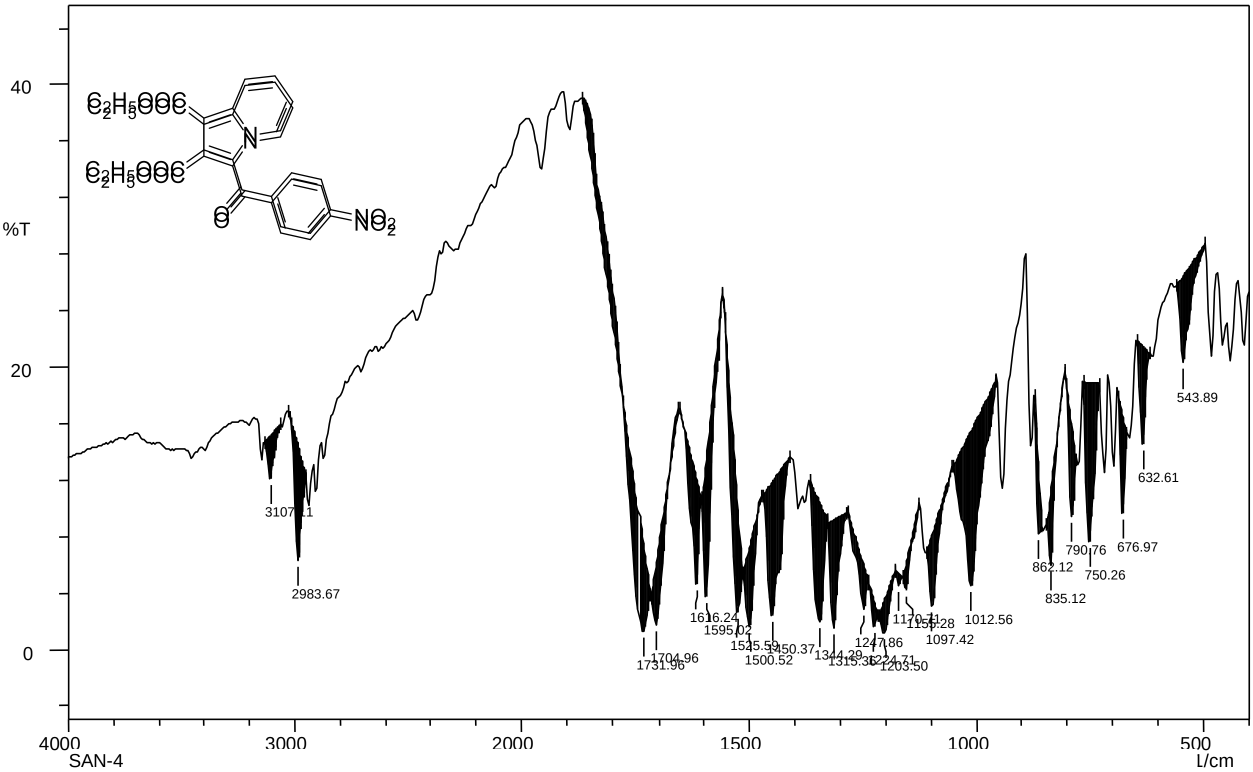

Figure S7: FT-IR of diethyl-3-(4-nitrobenzoyl)indolizine-1,2-dicarboxylate (**4c**)

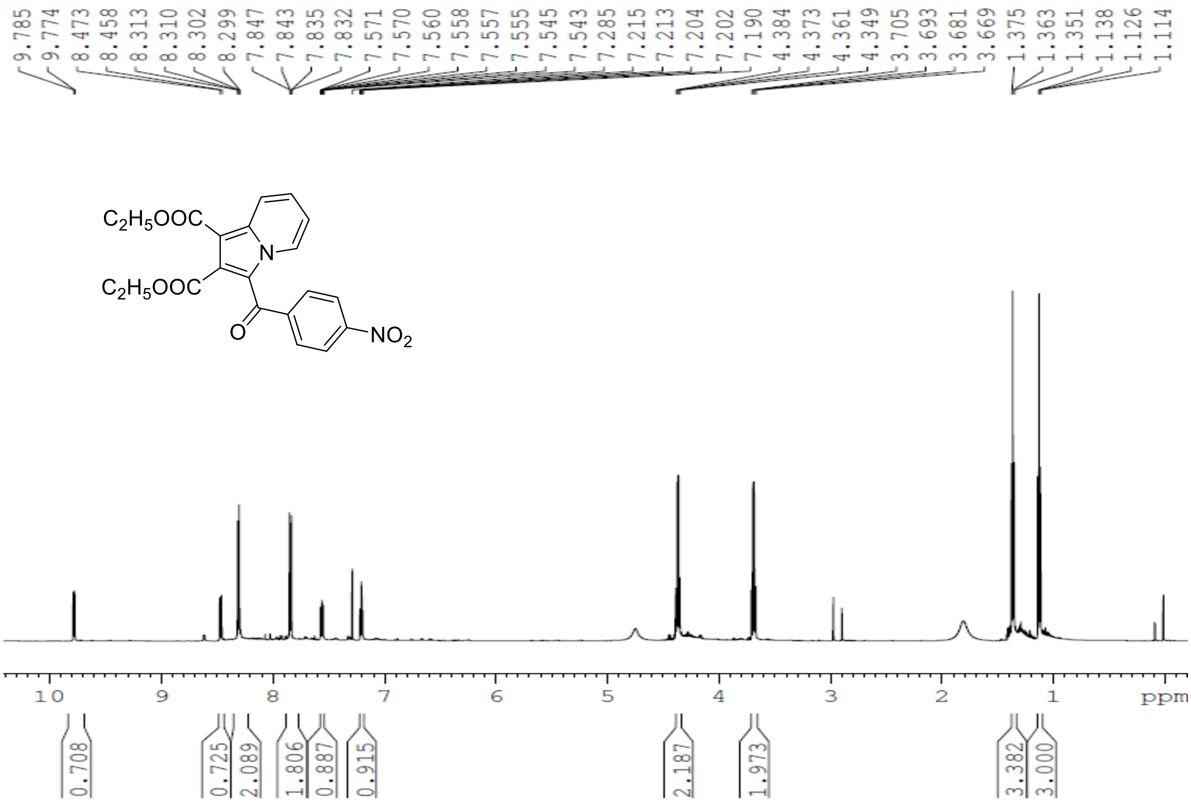

Figure S8: ^{1}H-NMR of diethyl-3-(4-nitrobenzoyl)indolizine-1,2-dicarboxylate (**4c**)

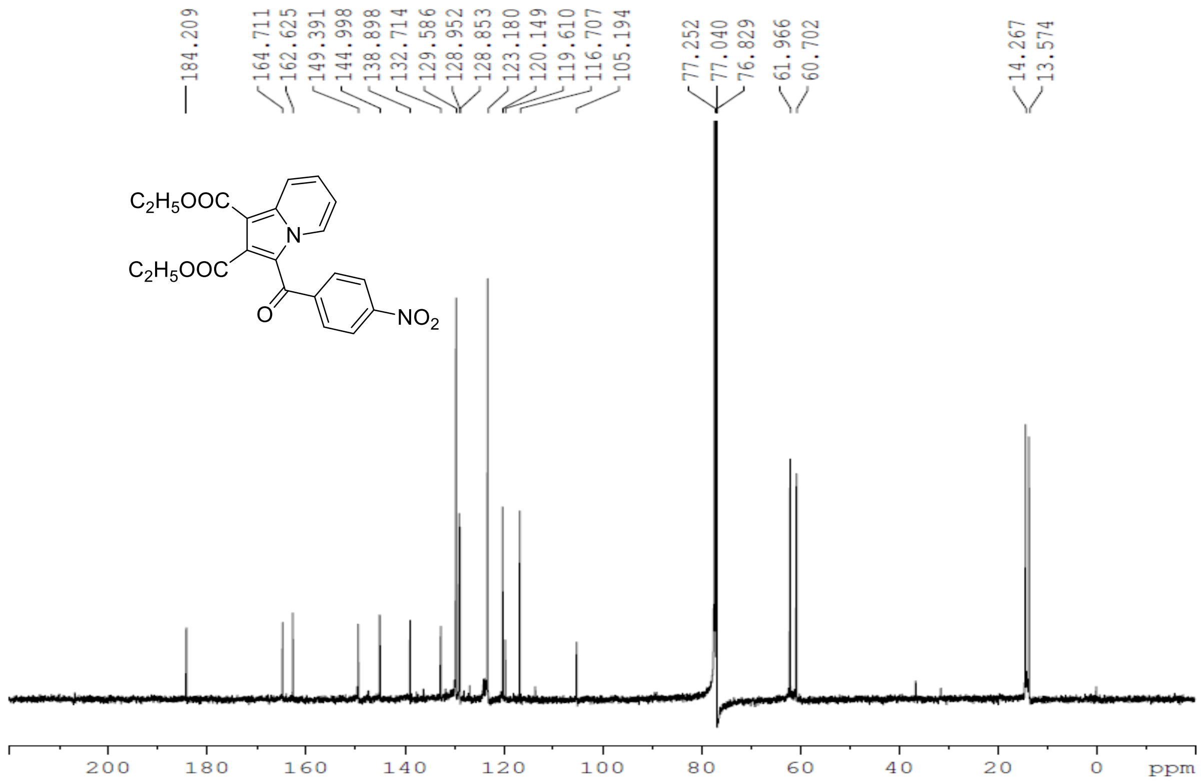

Figure S9: ^{13}C-NMR of diethyl-3-(4-nitrobenzoyl)indolizine-1,2-dicarboxylate (**4c**).

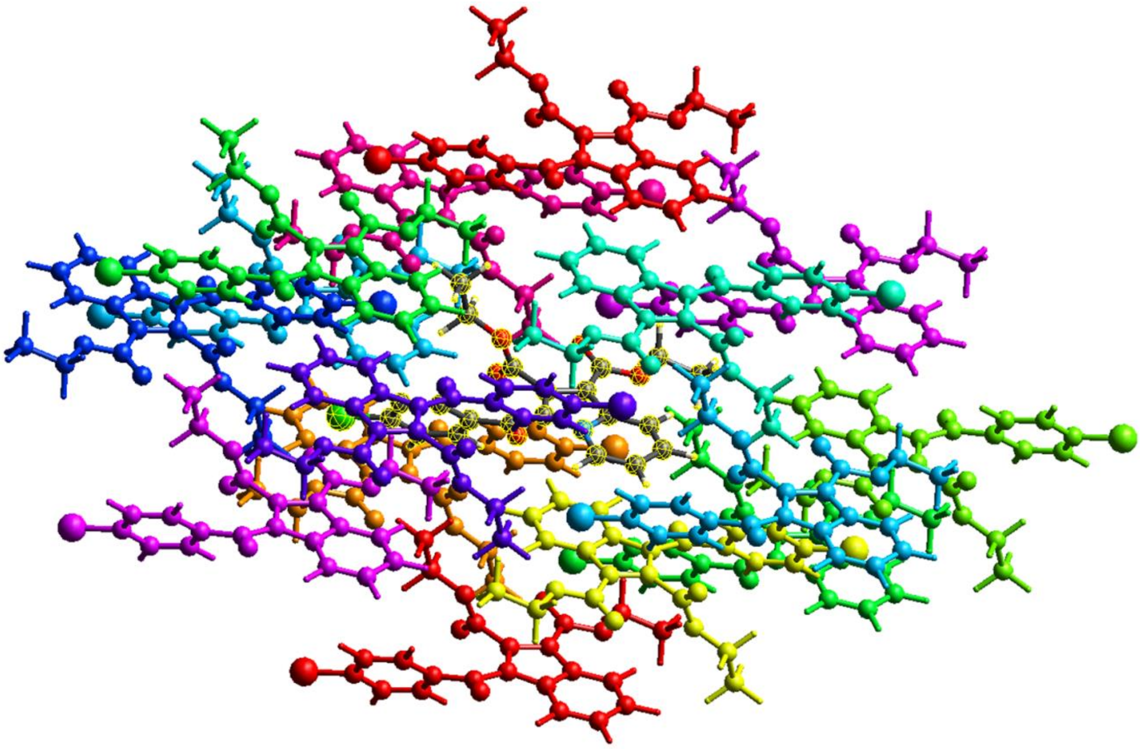

Figure S10. Energy frameworks corresponding to the total interaction energy between the selected molecule **4b** and the molecules present in a 3.8 A° cluster around it.

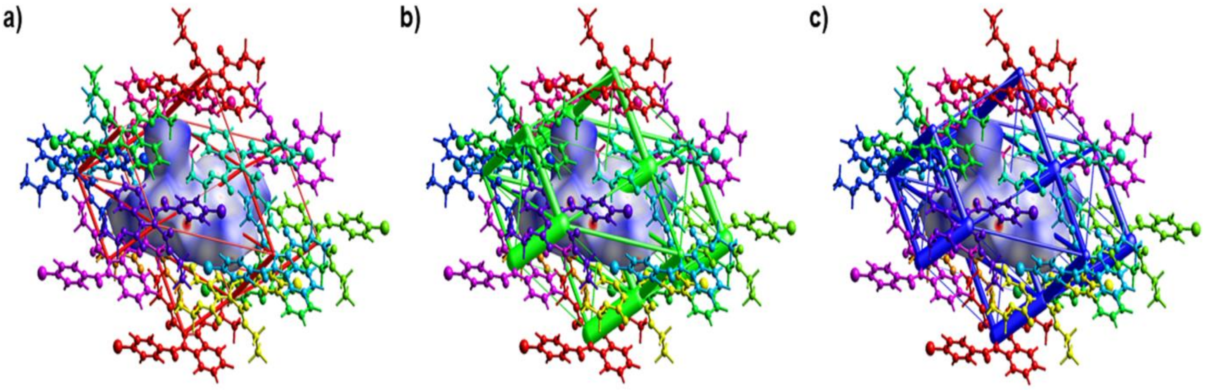

Figure S11. d_{norm} mapped on Hirshfeld surface of the molecule **4b** with energy framework in the form of **(a)** Coulombic energy, **(b)** dispersion energy and **(c)** total energy.

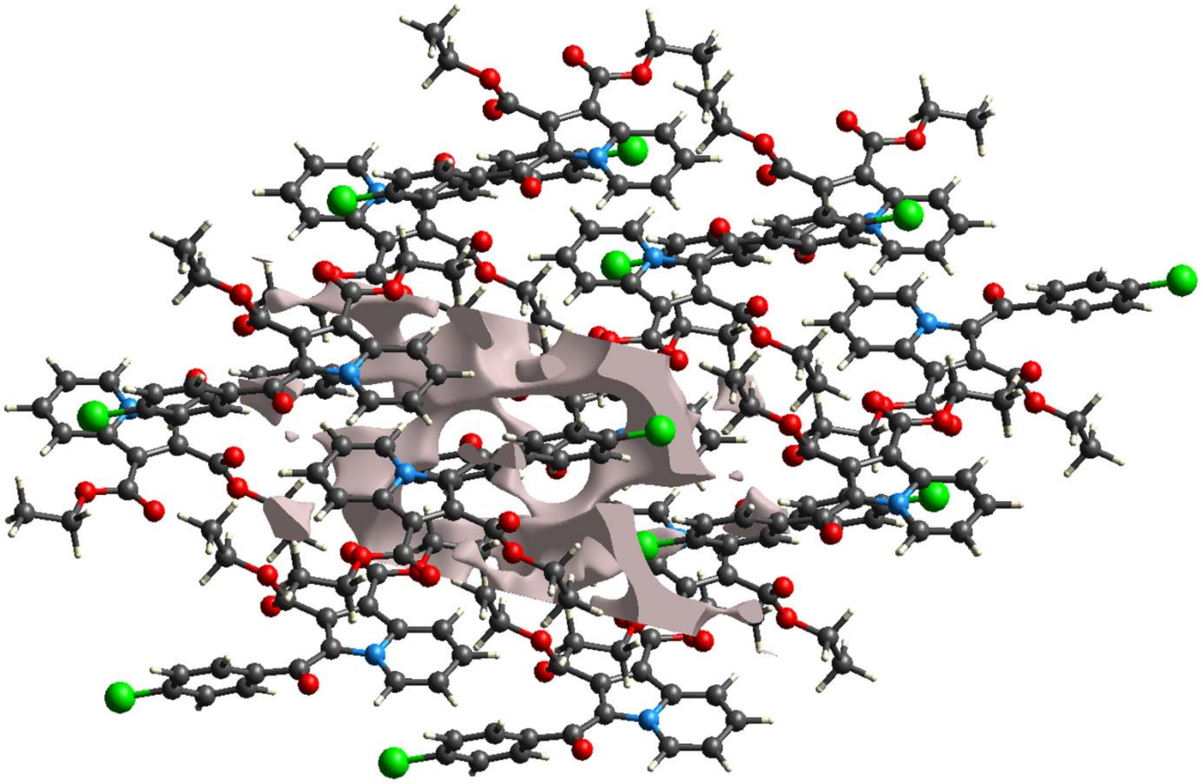

Figure S12. Crystal voids corresponds to promolecule surface including all the atoms in the title compound **4b.**

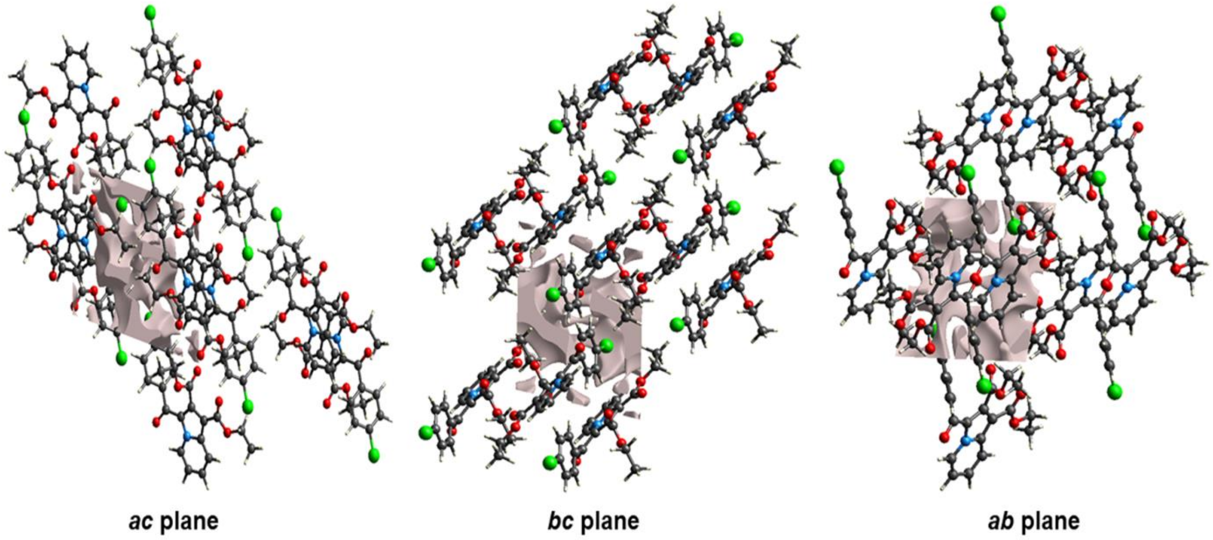

Figure S13. Crystal voids present in the title compound **4b** along with ***ac*** plane, ***bc*** plane and ***ab*** plane respectively.

References

[1] M.J. Turner, J.J. McKinnon, D. Jayatilaka, M.A. Spackman, Visualisation and characterisation of voids in crystalline materials, CrystEngComm, 13 (2011) 1804-1813.

[3] M. Ashfaq, M.N. Tahir, A. Kuznetsov, S.H. Mirza, M. Khalid, A. Ali, DFT and single crystal analysis of the pyrimethamine-based novel co-crystal salt: 2,4-diamino-5-(4-chloro-phenyl)-6-ethylpyrimidin-1-ium:4-hydroxybenzoate:methanol:hydrate (1:1:1:1) (DEHMH), Journal of Molecular Structure, 1199 (2020) 127041.

[3] A. Ali, M. Khalid, M.F.U. Rehman, S. Haq, A. Ali, M.N. Tahir, M. Ashfaq, F. Rasool, A.A.C. Braga, Efficient Synthesis, SC-XRD, and Theoretical Studies of O-Benzenesulfonylated Pyrimidines: Role of Noncovalent Interaction Influence in Their Supramolecular Network, ACS Omega, 5 (2020) 15115-15128.
